# Sex Chromosomes and Master Sex-Determining Genes in Turtles and Other Reptiles

**DOI:** 10.3390/genes12111822

**Published:** 2021-11-19

**Authors:** Dominique Thépot

**Affiliations:** Université Paris-Saclay, Université de Versailles Saint-Quentin-en-Yvelines (UVSQ), INRAE, BREED, 78350 Jouy-en-Josas, France; dominique.thepot@inrae.fr

**Keywords:** Genetic Sex Determination (GSD), lizards, snakes, squamates, homologous genes, RAD-seq, genome coverage

## Abstract

Among tetrapods, the well differentiated heteromorphic sex chromosomes of birds and mammals have been highly investigated and their master sex-determining (MSD) gene, *Dmrt1* and *SRY*, respectively, have been identified. The homomorphic sex chromosomes of reptiles have been the least studied, but the gap with birds and mammals has begun to fill. This review describes our current knowledge of reptilian sex chromosomes at the cytogenetic and molecular level. Most of it arose recently from various studies comparing male to female gene content. This includes restriction site-associated DNA sequencing (RAD-Seq) experiments in several male and female samples, RNA sequencing and identification of Z- or X-linked genes by male/female comparative transcriptome coverage, and male/female transcriptomic or transcriptome/genome substraction approaches allowing the identification of Y- or W-linked transcripts. A few putative master sex-determining (MSD) genes have been proposed, but none has been demonstrated yet. Lastly, future directions in the field of reptilian sex chromosomes and their MSD gene studies are considered.

## 1. Introduction

Sexual reproduction is ubiquitous in all vertebrates, and the formation and development of gonads, either testes or ovaries, is determined by the sequential action of members of the same genetic network. While the downstream actors of this network are well conserved, the elements triggering the process are surprisingly variable. In Environmental Sex Determination (ESD), the future sex of the gonad depends on environmental conditions. This environmental factor is generally the incubation temperature of the developing egg, and this peculiar case is known as Temperature Sex Determination (TSD). In Genetic Sex Determination (GSD), the bipotential gonad develops into either testis or ovary based on the control of a master sex-determining (MSD) gene upstream of the whole network. This MSD gene is located on only one member of a special pair of non-identical chromosomes called sex chromosomes. The sex with an odd number of MSD is the heterogametic sex, and the other is the homogametic sex. By convention, when male is the heterogametic sex, the male specific sex chromosome is named Y and the other X. When it is female, the female specific sex chromosome is called W and the other Z. The MSD gene can act in two different ways, either as male or female dominant gene on the Y or W chromosome respectively, or in a dose-dependent manner as a male gene on the Z chromosome or a female gene on the X chromosome.

**Figure 1:**
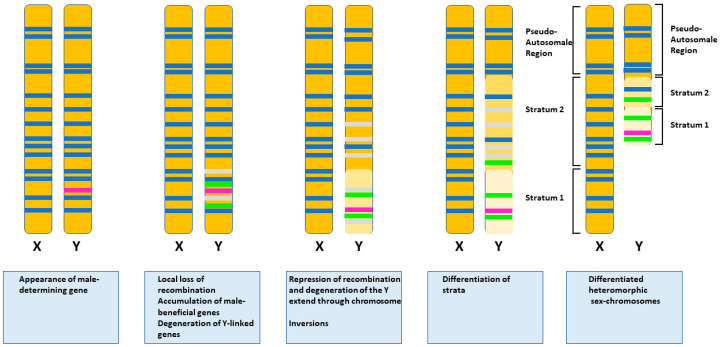
Current model for evolution of heteromorphic sex chromosomes from a pair of autosomes. Steps are shown from left to right. Ancestral genes are represented by dark blue lines, the male-determining gene is represented by a pink line, male beneficial genes are represented by green lines, and degenerating genes are represented by grey lines. Faded colours in the chromosome show the appearance of strata.

Apart from extremely rare cases [1], almost all sex chromosomes evolve from a pair of autosomes as demonstrated by the persistence of a homologous region, the pseudo-autosomal region, between the two members of the pair. This evolution begins with the appearance of an MSD gene, either by neofunctionalization or translocation, in one member of the pair, the proto-Y for example (Figure 1). Such changes have drastic consequences for the evolution of the proto-Y chromosome. For reasons not yet fully understood and highly discussed, suppression of recombination between the proto-X and the proto-Y occurs in the region of the chromosome surrounding the MSD [2]. From this point, the two proto-sex chromosomes continue to differentiate. This process includes the acquisition of sexually antagonistic genes, the degeneration of Y-linked genes, the accumulation of repetitive sequences, intrachromosomal rearrangements such as inversions, and the expansion of the non-recombining region. This continuing differentiation of the sex chromosomes may lead to the appearance of morphologically distinct chromosomes called heteromorphic sex chromosomes. Before they become morphologically distinguishable by classic cytogenetics, they are homomorphic. This is just a visual criterion, not a functional one. As a consequence, there is no linear relation between the degree of genetic differentiation between sex chromosomes and the fact that they are homomorphic or heteromorphic. Homomorphic chromosomes can be as genetically differentiated and evolutionary old as heteromorphic chromosomes. Thus, if heteromorphic chromosomes are evidence of GSD, the opposite is not true, and GSD can occur within homomorphic chromosomes. Because the non-recombining region expands stepwise across the proto-Y chromosome, strata appear in the Y-chromosome. In each stratum, the remaining ancestral autosomal genes have started to diverge from their chromosome X counterparts at the same time. These pairs of genes are called gametologs. The degree of divergence between gametologs can be assessed by the measure of the synonymous nucleotide substitution rate (dS or Ks) between them. So, each stratum is characterized by the Ks of its gametologs. The MSD gene is located in the oldest strata and has high Ks.

Until now, only a few MSD genes have been identified in vertebrates. Their comparative study reveals the use of a restricted set of genes of the sex determination network that are likely to evolve as MSD genes. Several times, independently according to phyla, certain genes have been recruited by evolution, either in their native form or in the form of paralogs, to become the major gene for determining sex in a given species or phylum. For instance, two paralogs of *Sox3*, *SRY* in marsupials and placental mammals [3,4], and *Sox3Y* in the fish *Oryzias dancena* [5], have independently acquired this role. Similarly, *Dmrt1* is probably the major gene for sex determination in birds [6,7] and fish *Cynoglossus semilaevis* [8]. Two of its paralogs (*DmW* and *Dmrt1Y*) play this role, respectively, in the frog *Xenopus laevis* [9] and two species of fishes, the medaka *Oryzias latipes* [10,11] and *Oryzias curvinotus* [12]. In fish, paralogs of the *Gsdf* gene (*GsdfY* in *Oryzias luzonensis* [13]), *Gdf6* gene (*Gdf6aY* in *Nothobranchius furzeri* [14], *B-Gdf6b* in *Astyanax mexicanus* [1], *Amh* (Antimullerian hormone) gene (*AmhY* in *Odontesthes hatcheri* [15], *Amhby* in the pike *Esox lucius* [16], *Amhy* in *Gasterosteus aculeatus* [17]) or its receptor *AmhR* gene (*Amhr2* in the Fugu *Takifugu rubripes* [18], *Amhr2Y* in the Canadian perch *Perca flavescens* [19], *amhr2bY* in ayu *Plecoglossus altivelis* [20]) have also become MSD genes. Finally, *Amh* is probably the major gene for determining sex in monotreme mammals [21,22]). It should also be added that this persistence of the same actors in the sex determination network, associated with a great variability of the MSD gene according to the phylum, had already been noted in insects, thus leading to the formula “Masters change, slaves remain” [23]. It is notable, however, that this rule is not absolute and that MSD genes can also be recruited outside the classic members of the network. This is the case in the rainbow trout *Oncorhynchus mykiss* where *SdY*, a paralog of *Irf9*, a gene regulating interferons, has taken on this function [24]. *SdY* interacts directly with *FoxL2*, a major gene for pushing gonad differentiation towards the female pathway, to inhibit the aromatase promoter (*Cyp19A1*) and therefore the synthesis of oestrogens [25]. Two other examples are known in the catfish *Ictalurus punctatus* [26] and the amphibian axolotl [27]. In catfish the MSD gene is a male specific isoform of the gene *BCAR1* (Breast-Cancer AntiResistance 1), an adaptor protein expressed in all tissues, which acts by binding to the alpha oestrogen receptor and therefore by inhibiting the action of oestrogens [26]. In the axolotl *Ambystoma mexicanum* the MSD gene is a duplication of the *ATRX* gene (a helicase domain protein, involved in the deposition of specific histones on repeated sequences) called *ATRW*, which is specific to chromosome W [27]. The mechanism of action by which *ATRW* would push differentiation from the gonad towards the female pathway is completely unknown at this time. However, *ATRX* is well known for its role in mammalian sexual differentiation, as demonstrated by the various degrees of gonadal dysgenesis observed in human XY mutated for *ATRX*, the most drastic phenotype being a complete male to female sex reversal [28].

This list of species with identified MSD genes includes no reptilian species so far. There are several reasons for this delay. TSD was discovered in reptiles [29,30] and is a specific feature of this group among tetrapods. During the last century, it has attracted more attention from researchers in the field of reptile reproduction to the detriment of GSD. Moreover, many reptiles lack heteromorphic chromosomes, the easiest way to discover GSD, and this fact has led to the underestimation of GSD occurrence in reptiles. Genetic manipulation of embryos was unavailable in reptiles, and as no reptiles have high economic value there was no financial interest to push the research in developing reptile-specific techniques. All these reasons explain why the identification of MSD genes in reptiles still lags behind research in other groups. However, with the development of new technology, this gap is beginning to be filled and incredible progress has been accomplished during the last two decades. However, the level of knowledge is still very heterogeneous from one family to another, ranging from almost nothing to the identification of a restricted set of candidate genes.

The aim of this article is to summarize the current knowledge in each group of reptiles with a focus on turtles and less detail on squamates. In the first section, basic information will be provided for each group, concerning its phylogenic position, the occurrence of TSD or GSD, the presence or absence of heteromorphic sex chromosomes, the degree of differentiation of its sex chromosomes, the homology of sex chromosomes with chicken chromosomes, and putative MSD candidate genes. In the second section, some points about the transitions between TSD and GSD, or the repetitive selection of the same chromosomic region to become sex chromosomes will be discussed, before addressing the question of how recent methods will help to identify MSD genes in reptiles.

## 2. Overview of GSD in Reptiles

### 2.1. Turtles

**Figure 2:**
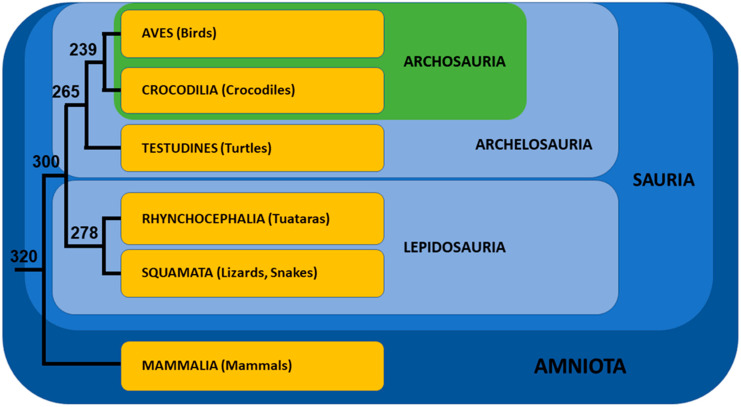
Phylogenic tree of amniotes. Relative position and grouping of each clade are shown. Branch lengths are not proportional to time. Numbers indicate divergence time (Myrs) from present for nodes, according to [38]. Colors indicate the different level of clades in the phylogenetic tree.

The phylogenetic position of Turtles among Reptiles (=Sauria) had been a matter of controversy for a long time (see [31] for review), but the development of molecular phylogenetics clarified the situation at the beginning of the century. Molecular studies using ultraconserved elements [32], mitochondrial and nuclear genes [33,34], and finally the sequences of three turtle genomes [35,36] led to the consensus that turtles (=Testudines) are the sister-group of Archosauria (=Birds + Crocodilians). Together, they form Archelosauria [37], the sister group of Lepidosauria (=Tuatara + Lizards + Snakes) (Figure 2).

**Figure 3:**
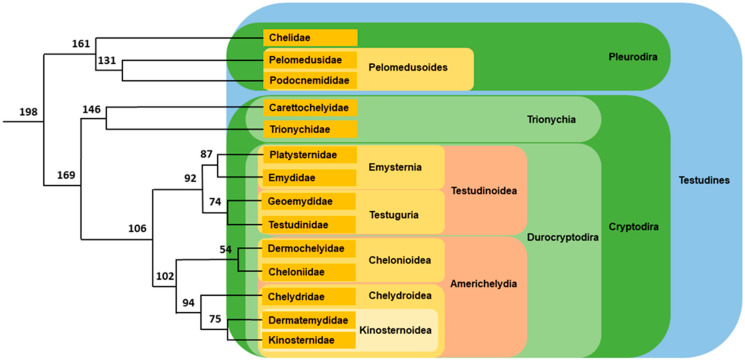
Phylogenic tree of turtle families. Relative positions of each turtle family and superior clade are represented. Branch lengths are not proportional to time. Numbers indicate divergence time (Myrs) from present for nodes, according to [39]. Colors indicate the different level of clades in the phylogenetic tree from the fourteen families (golden yellow) to the order (blue).

Phylogenomic analyses highlighted the evolutionary history and relative phylogenetic relationships among turtle families [37,39,40,41]. The first division occurred in the early Jurassic period. It separated the suborder Pleurodira, or side-necked turtles, that originated in Gondwana from the suborder Cryptodira, or hidden-necked turtles, with a northern origin located in Laurasia. Pleurodira contains three families, all distributed in the southern hemisphere. Cryptodira is divided into Trionychia, soft-shelled turtles, itself with two families, and Durocryptodira with nine families dispatched in two clades, Testudinoidea and Americhelydia (Figure 3). This phylogenetic tree is robust and widely accepted. In almost each family, the relative position of the different genera and even those of the different species is now well established.

Among Archelosauria, the turtles and the two clades of Archosauria show contrasting sex-determining mechanisms. All known Crocodilians are temperature sex-determined species [42], whereas the same ZW genetic sex-determining system is present in all bird species [43]. Slight modifications of the system exist in some birds, such as a multiple sex chromosome system in the Adélie penguin [44]. Another example is the presence of a neo-sex chromosome formed by the fusion of the Z chromosome with part of chromosome 4 alone or in combination with parts of chromosomes 3, 4 and 5 in Sylvioidea passerines [45]. The *DMRT1* gene, located on the Z chromosome and present in one copy in females and two copies in males, is generally thought to be the sex-determining gene in birds [6,7]. However, the absence of Z0 birds to be studied and the phenotype of ZZW birds [46] leave room for the possibility of an ovary-determining gene on the W chromosome (see [47] for review). In contrast, turtles exhibit great variation in sex determination systems with TSD species, GSD species with XY chromosomes and GSD species with ZW chromosomes. As most species of turtles are TSD species, it is thought that TSD is the ancestral state in turtles and that GSD systems arose several times independently during turtle evolution [48,49,50].

#### 2.1.1. Pleurodiran Turtles

**Figure 4:**
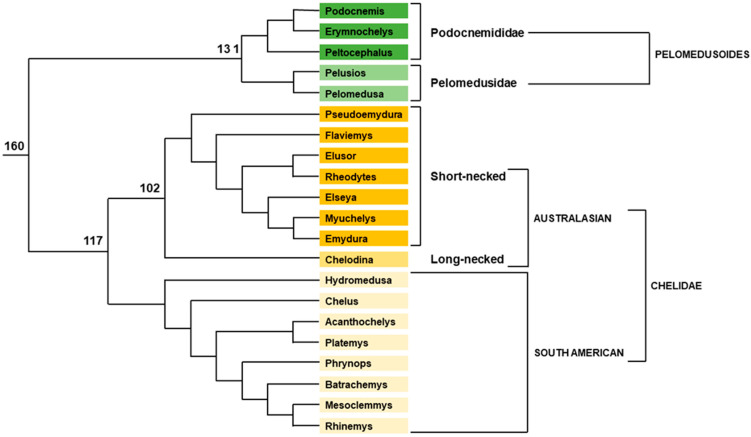
Phylogenic tree of pleurodiran turtles. Relative positions of each genus and superior clade are represented. Branch lengths are not proportional to time. Numbers indicate divergence time (Myrs) from present for some key nodes, according to [39]. Color of each genus indicates the different clades.

In Pleurodira, the superfamily Pelomedusoides is divided into two families. The first one, Pelomedusidae, encompasses African species dispatched into two genera: *Pelomedusa* with only one species recently split into 10 species [51] and *Pelusios* with 15–20 species [52]. The second, Podocnemididae, is composed of six South-American *Podocnemis* species, the South-American *Peltocephalus dumerilianus* (Schweiger, 1812) and the Madagascan *Erymnochelys madagascariensis* (Grandidier, 1867)). Among Podocnemididae, *Peltocephalus* is the sister group of *Erymnochelys* and *Podocnemis*, [53,54] (Figure 4). Karyotypes of Pelomedusoides examined so far (4 *Pelusios* species, *Pelomedusa subrufa* and all Podocnemididae species) show no detectable sex-chromosomes [55,56,57,58,59,60,61,62,63]. Moreover, TSD was demonstrated in one species of *Pelusios* [48], one species of *Pelomedusa* [48], *Podocnemis expansa* [64,65], *Podocnemis unifilis* [66], *Podocnemis erythrocephala* [67] and *Podocnemis lewyana* [68,69]. Another species, *Podocnemis sextuberculata*, was also cited as TSD species [67], but the studies leading to this statement are yet to be published. From field data, *P. dumerilianus* was first claimed to be a GSD species [70], but was later demonstrated to possess temperature-dependent sex determination [67,71]. Lastly, *E. madagascariensis* has TSD [72], but unfortunately this has not yet been published in peer-reviewed literature. It is thus generally accepted that TSD occurs in all Pelomedusoides species.

The situation is quite different in Chelidae, the last family in Pleurodira, which is divided between two clades: 23 South American species and around 35 Australasian species (Figure 4). All species tested so far by controlled incubation temperature experiments turned out to be GSD species. This includes the South American *Mesoclemmys gibba*, *Phrynops geoffroanus*, and *Phrynops hilari* [73], and the Australasian *Emydura macquarii* [74] (under the name *E. signata*); [75], *Chelodina longicollis* [76], *Elusor macrurus* [77], *Emydura subglobosa* and *Elseya novaguinae* [73]. However standard karyotypes of most species from different genera (*Batrachemys*, *Chelus*, *Hydromedusa*, *Mesoclemmys*, *Phrynops*, *Platemys*, *Chelodina*, *Elseya*, *Emydura*, *Pseudoemydura*, *Rheodytes*), showed undifferentiated homomorphic sex chromosomes [58,78,79,80,81]. Heteromorphic sex chromosomes were only described in *Acanthochelys radiolata* (under the name *Platemys radiolata*) with a heteromorphic pair of one metacentric and one acrocentric chromosome [82]. As there are five pairs of subtelomeric or acrocentric chromosomes in other *Acanthochelys* species and only four pairs in *A. radiolata*, this heteromorphic pair could be sex chromosomes that evolved from an acrocentric pair. However, only one male was studied with no female for comparison, and therefore other explanations are possible, such as chromosomal polymorphism. Moreover, in the three other *Acanthochelys* species, only female karyotypes have been described and the male karyotype is unknown [82]. Thus, the presence of heteromorphic sex chromosomes in *A. radiolata* and perhaps in other *Acanthochelys* species needs to be confirmed by the study of more animals of both sexes. Another potential sign of sex chromosome differentiation occurs in *Chelus fimbriatus* [78]. At the pachytene stage during male meiosis, one bivalent chromosome presents a region without pairing. Such an image could suggest the presence of an intrachromosomal inversion in one chromosome of the pair, and it is tempting to speculate that it is the first sign of Y chromosome differentiation. However, only one male was studied, and this result must be replicated and confirmed by the identification of Y-specific genetic markers, which are still lacking today. The occurrence of sex macrochromosomes in South American Chelids is thus still uncertain.

More results were obtained with the development of a new molecular cytogenetic approach: comparative genome hybridization (CGH). This method consists of independent labelling of male and female genomic DNA with two different fluorescent molecules (green and red) before cohybridization of male or female metaphasic chromosome spreads. Male or female specific parts of the sex chromosomes are thus labelled green or red, while autosomes and the pseudo-autosomal part of the sex chromosomes are labelled yellow. Using CGH allowed the identification of male-specific regions of the Y chromosome in several Australasian species: *C. longicollis* [83], *E. macquarii macquarii* [84], *E. subglobosa* [85], *E. macquarii krefftii*, *E. novaguinae*, *Chelodina rugosa*, *C. expansa*, *C. reimanni*, *C. novaeguineae*, and *C. mccordi* [86]. Interestingly the sex chromosome is a difficult to detect minichromosome in long-necked *Chelodina* (2n = 54), whereas it is the fourth largest macrochromosome in its sister-group, the Australasian short-necked Chelids (*Emydura* and *Elseya*) (2n = 50). Different scenarios for the sex chromosome evolutionary history in these species have been proposed [84,85,86,87]. They range from independent origin of sex chromosomes in *Chelodina* and *Emydura* to shared origin with either an ancestral sex minichromosome fused with a macrocromosome in *Emydura* or the translocation of the sex-determination region from an ancestral sex macrochromosome to a sex minichromosome in *Chelodina*. Because it had been suggested in Podocnemididae that the presence of interstitial telomeric repeats (ITR) may be the remnants of ancient events of (mini)chromosome fusions [88], looking for such ITRs in the *Emydura* Y-chromosome could have helped discern whether the fusion or the fission scenario was correct. However, it was later shown that ITRs were rare in turtles and not correlated with interchromosomal rearrangements [89]. Whatever the sex chromosome ancestral state and evolution might be, the *Emydura* Y-chromosome probe paints not only the entire fifth largest autosome of *C. longicollis*, but also part of its Y chromosome [87]. Therefore, both Y-chromosomes share some homologous sequences. Even though this provides no definitive evidence they carry the same sex-determination locus, it is a reasonable hypothesis. The recent study by CGH and C-banding of the South American *Rhinemys rufipes* allowed the discovery of a Y minichromosome in this species [90]. The sex minichromosome pair is the tiniest and the Y chromosome is highly heterochromatic. Interestingly, the tiniest minichromosome pair of a male *Phrynops hogei* exhibits the same pattern, with one member of the pair being highly heterochromatic and the other one not at all [80], suggesting the two species share the same XY sex minichromosome system. However, only one male of *P. hogei* (and no female) was studied, and more animals of both sexes need to be analysed before any conclusion can be drawn. The authors of the *R. rufipes* study suggest the possibility that a sex minichromosome is the ancestral state in Chelidae [90]. They do not take into account the fact that both *Acanthochelys* and *Chelus* perhaps have sex macrochromosomes, and that, therefore, the ancestry of sex minichromosome in South American Chelidae is far from being established. Moreover, the homology between *Chelodina* and *Rhinemys* sex minichromosomes remains an open question until otherwise proven.

To date, neither the synteny of the Y-minichromosome with chromosomes of other species nor its gene content are known. Genetic markers allowing comparison between *Emydura*, *Chelodina* and South American Chelid sex chromosomes are still lacking. Thus, the unicity of GSD mechanism in Chelidae remains to be demonstrated. If all Australasian Chelids share the same XY system, it will be at least 102 million years old [39]. If this is the case for all Chelids, the common XY system will be between 117 and 160 million years old [39]. The master sex-determining gene is still to be found, but some candidates can already be ruled out. Bacterial Artificial Chromosomes (BACs) carrying *Chrysemys picta* (another turtle) sequences from genes involved in the sex determination network were used to localize homologous genes on the chromosomes of different turtle species [91]. The tested genes were *Dax1*, *Dmrt1*, *Fgf9*, *Fhl2*, *Foxl2*, *Gata4*, *Rspo1*, *Sf1* (=*Nr5a1*), *Sox9* and *Wt1*. The ten probes gave signals on *Emydura* autosomes, but none of them labels *Emydura* sex chromosomes [91]. Consequently, these genes can be excluded as candidates for sex determination locus in *Emydura*.

To summarize, all Chelids seem to possess a GSD system of sex determination with poorly differentiated XY chromosomes that can be identified only by high resolution cytogenetics. Australasian species likely share a common sex determination system involving an unknown master gene, whereas South American species remain in urgent need of investigation with modern methods.

#### 2.1.2. Cryptodiran Turtles

##### Trionychia

**Figure 5:**
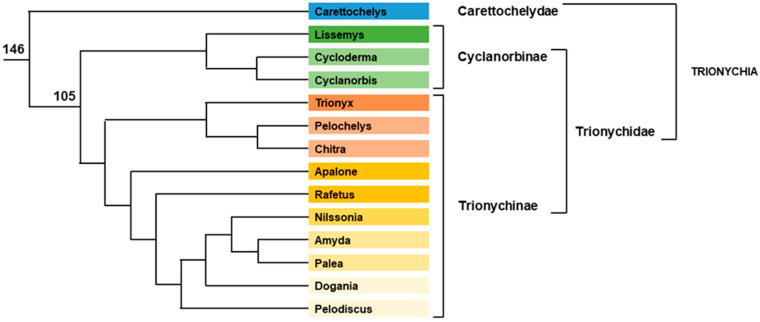
Phylogenic tree of trionychid turtles. Relative position of each genus and superior clade are represented. Branch lengths are not proportional to time. Numbers indicate divergence time (Myrs) from present for some key nodes, according to [39]. Color of each genus indicates the different clades.

The New Guinean and Australian pig-nosed turtle *Carettochelys insculpta* is the sole member of the Carettochelyidae family. Temperature sex determination was demonstrated experimentally in this species [92]. The Trionychidae family (softshell turtles) is composed of 30 species dispatched in two subfamilies, Trionychinae with 24 species and Cyclanorbinae with 6 species (Figure 5). The history of the Trionychidae sex-determination discovery shares some similarities with that of Chelidae. The standard karyotypes (2n = 66 for all species tested so far) show homomorphic chromosomes [93,94,95], while controlled incubation temperature experiments recover GSD species, *Apalone spinifera* (under the name *Trionyx spiniferus*) [96], *Pelodiscus sinensis* (under the name *Trionyx sinensis*) [97] and *Apalone mutica* [48,98]. As for Chelids, CGH clarified the situation, demonstrating the existence of a sex minichromosome pair ZW in *P. sinensis* [99]. It also definitively ruled out some erroneous claims about possible TSD in some *P. sinensis* populations [100]. Nuclear Organizer Region (NOR) and 18S-28S rRNA clusters are located on this minichromosome [99], with the differentiated W chromosome more strongly stained by such probes than the smaller Z chromosome. The larger size of the W chromosome is partly due to the amplification of the rRNA cluster at its centre. W chromosome differentiation also implies a paracentric inversion compared to the Z chromosome [101]. Later, such a NOR-bearing sex minichromosome pair was also discovered in *A. spinifera* [102] with the same larger W chromosome. ZW sex chromosomes share homology with chicken chromosome 15 [101], even though the order of the genes is not the same. The genome assembly of P. sinensis was the first turtle genome available [36], and the sequenced animal being a female, it contains both Z and W sequences. However, the sex chromosome sequences are still unmapped, and a physical map of the sex chromosomes is still lacking. Using the linkage with chicken chromosome 15, six markers of *P. sinensis* chromosome Z were developed [103]. qPCR experiments dosing the gene copy number in both male and female genomic DNA allowed testing of *P. sinensis*, *A. spinifera* and eight other Tryonichids from genera *Lissemys*, *Chitra*, *Amyda* and *Nilssonia* with success [103]. Female/male ratio of 0.5 indicated that the gene was Z-specific, whereas a ratio of 1 indicated the gene was autosomal or pseudoautosomal. All the species were found to possess a homologous Z chromosome [103]. This suggests that the common ZW system arose after the split between Carettochelyidae and Trionychidae (200 Myr) and before the split between Trionychinae and Cyclanorbinae (120 Myr). Chicken chromosome 15 is a small chromosome bearing less than 400 protein-coding genes, but despite this and the availability of the genome, the master sex-determining gene is still unknown in the family. As for Chelids, ten candidates were ruled out because none of them localized on *P. sinensis* Z or W chromosomes. However, *Sf1* and *FoxL2* mapped on Z and Y chromosome in *A. spinifera* but not in *P. sinensis* [91], demonstrating that Z chromosome content slightly varies in different species of Trionychidae. Two recent studies addressed the questions of the Z chromosome content in genus *Apalone* and of the presence or absence of gene dosage compensation [104,105]. By comparing the read coverage between male and female genomic sequencing in *Apalone ferox*, selecting genes with a female to male ratio less than 0.7, and with orthologs on chicken chromosome 15 (GGA15), 220 candidate Z-specific genes were identified [104]. The adult blood cell mRNA expression level of these genes has been evaluated. Among the 220 candidate Z-specific genes, the expression level of 102 genes could be compared between males and females. Their female to male ratio is about half those of autosomal genes, demonstrating there is no dosage compensation between males and females in this species [104]. Moreover, using the expression level in *Caretta caretta* (a TSD species of another family) as a control, the authors concluded that expression of Z-specific genes was similar to the ancestral autosomal level in males, but lowered in females. Thus, they concluded there is no global gene dosage compensation in *A. ferox*. The second study was performed in a closely related species, *A. spinifera* [105]. Low-coverage genome sequencing of one male and one female and an analysis of read depth coverage after mapping of the reads against the *P. sinensis* genome identified putative sex chromosome scaffolds in this species. A total of 2.6 Mb of W-specific sequence and 34.2 Mb of Z-specific sequence was recovered, and the scaffold uncovering the pseudoautosomal boundary was identified [105]. After filtering with different criteria (homology with GGA15 genes, percentage of sequence identity, etc.), a final set of 245 Z-linked genes was established, and their RNA-seq SNP analysis revealed 119 genes without SNPs in females. The expression of these 119 Z-linked genes in different organs and at different developmental stages was compared with those of 388 *A. spinifera* autosomal genes, and with orthologs of both these Z-linked and autosomal genes in *Chrysemys picta*, a TSD species of another family. RNA samples were collected from adults (liver and gonads), hatchlings (blood, liver, and gonads) and embryos at five different developmental stages and incubated at two different temperatures. The analysis revealed a complex pattern of dosage compensation to equilibrate expression of sex-linked genes compared to their ancestral homologs. This dosage compensation was thermosensitive, more local than global and varied by tissue and developmental stage [105].

##### Durocryptodira

Durocryptodira is divided in two clades: Americhelydia and Testudinoidea (Figure 3).

Americhelydia

Americhelydia includes two clades. The first one, Chelonioidea, contains all sea turtles, separated into two families: Dermochelyidae, with the only leatherback sea turtle (*Dermochelys coriacea*), and Cheloniidae, with six species in five genera. Temperature dependent sex-determination occurs in all sea turtle species: *D. coriacea* [106], *Chelonia mydas* [107], *Eretmochelys imbricata* [108], *Natator depressus* [109], *C. caretta* [110], *Lepidochelys olivacea* [111] and *Lepidochelys kempii* [112].

The second clade, Chelydroidea, is divided into three families: Chelydridae with only two genera, Chelydra and Macrochelys, which exhibit TSD [48,113], Dermatemydidae with the sole species *Dermatemys mawii* also demonstrated TSD [114], and Kinosternidae, for which the situation is more complicated. Kinosternidae are divided in two subfamilies with different chromosomal numbers: Kinosterninae (2n = 56), which contains the genera *Kinosternon*, with at least 20 species, and *Sternotherus* with four species, and Staurotypinae (2n = 54) with the genus *Staurotypus*, which includes two species and *Claudius angustatus*. Kinosterninae and Staurotypinae diverged 55 million years ago, Staurotypus and Claudius 25 million years ago, and the two Staurotypus species 5 million years ago [39]. In all species of Kinosterninae tested so far (3 *Sternotherus* and 10 *Kinosternon*), TSD occurs [48,73,115]. In contrast, the sex ratio following egg incubation at different temperatures demonstrated GSD in *Staurotypus* [48,114] and less convincingly in *Claudius* because of the sample size (61 versus 7 only) for the two tested temperatures [114]. Moreover, both *Staurotypus salvinii* and *S. triporcatus* possess heteromorphic XY chromosomes [116], whereas *C. angustatus* exhibits homomorphic, supposed XY, sex chromosomes [116]. This cast some doubt on the reality of GSD in Claudius, which remains to be more firmly established. There is a slight difference between the two *Staurotypus* species. In both species the Y-chromosomes are acrocentric and smaller than the X-chromosomes, whereas the X chromosomes are acrocentric in *S. triporcatus* and subtelomeric in *S. salvinii*. Using *P. sinensis* chromosome 6 as a probe to paint metaphase of S. triporcatus cells, Matsuda’s laboratory demonstrated that *S. triporcatus* sex chromosomes are homologous to *P. sinensis* chromosome 6, itself homologous to chicken Z chromosome [117]. Moreover, they cloned 16 *S. triporcatus* homologs of chicken Z-chromosome genes and used them in FISH experiments. They mapped them on the X and Y chromosomes of both *Staurotypus* species, demonstrating the conservation of the gene order between *Staurotypus* sex chromosomes and ostrich Z chromosome, which represents the ancestral state of the avian Z chromosome [117]. Every 16 probes mapped on both X and Y chromosomes in the same order, suggesting these chromosomes are still in a poorly differentiated state and have not yet suffered major rearrangements, such as inversions. Among these 16 genes, *Dmrt1* is of special interest because of it is pivotal in the male sex determination pathway in vertebrates, and itself or its paralogs have been selected by evolution to become the master sex-determining gene in birds, *Xenopus laevis* and some fishes. *Dmrt1* could thus appear as a good candidate for such a role in Staurotypus, but it raises serious issues. Even if it could be due to the limits of the techniques used, *Dmrt1* seems to be present in the pseudoautosomal region of both X and Y chromosomes, and not in the differentiated region. Moreover, two groups independently cloned *S. triporcatus Dmrt1* from both male and female tissues and found the same sequence [117,118,119]. Therefore, both males and females seem to have two copies of the same *Dmrt1* gene, even if the lack of Y-specific *Dmrt1* could be due to sequence divergence in the region where the primers for amplification have been chosen. It is thus difficult to understand how *Dmrt1* could be responsible for the sex determination in *Staurotypus*. A provocative hypothesis is that *Dmrt1* is not the sex-determining gene. In fact, none of the 16 *S. triporcatus* homologs of chicken Z-chromosome genes lie in the differentiated parts of either sex chromosomes, and the *P. sinensis* chromosome 6 probe does not paint these differentiated regions [117]. Although chromosomes 1, 2, 3 and 5 are homologous between *S. triporcatus* and its close Kinosterninae relative *Sternotherus odoratus*, the *S.triporcatus* sex chromosome pair, the fourth in size, is homologous to *S. odoratus* chromosome 7 [91]. Therefore, a fusion of *S. odoratus* chromosome 7, bearing *Dmrt1*, with one of the minichromosomes could have resulted in the *S.triporcatus* sex chromosome, differentiating the region homologous to the minichromosome, where the true sex determining gene would occur. Only whole genome sequencing of *Staurotypus* will answer this issue. The other nine candidate genes, mapped to *S. triporcatus* chromosomes by [91], lie on autosomes and can be ruled out. The master sex-determining gene in Staurotypinae thus remains unknown.

It has been claimed that the X chromosome, and not the Y chromosome, is the evolutionary derived chromosome in *Staurotypus* [120], but this is questionable. Firstly, it is based on comparisons with karyotypes of distant turtle species and not that of its nearest relative *Claudius*, and on an erroneous phylogenic hypothesis [93]. Secondly, it disagrees with the previous statement that “*Claudius angustatus* karyotype is indistinguishable from that of female *Staurotypus*” [116] which suggests that the X chromosome is the ancestral state similar to the one existing in *Claudius*. Thirdly, the difference between the X and Y chromosomes in *Staurotypus* is partly due to differences in the copy number of the 18S–28S rRNA genes [117]. No available data on Claudius sex chromosomes allows to assess whether the X chromosome has been amplified or whether the Y chromosome has lost 18S–28S rRNA genes copies in *Staurotypus* compared to *Claudius*. There is, thus, a need for information about *Claudius* sex chromosomes before reaching a conclusion about the evolution of sex chromosomes in Staurotypinae.

Testudinoidea

Testudinoidea is divided in two clades, Testuguria and Emysternia which split one from each other 95 million years ago [39]. Testuguria is one of the less studied groups of turtles and for numerous genera the control of sex determination is still unknown. It includes two families: the Geoemydidae with 71 species, and the terrestrial Testudinidae with 65 species. No sex chromosomes have been described in Testudinidae, and all Testudinidae tested so far possess TSD. They belong to the genera *Astrochelys* [121], *Centrochelys* [122], *Chelonoidis* [123], *Gopherus* [124,125,126], *Malacochersus* [73], *Manouria* [127] and *Testudo* [30,128]. The genus *Aldabrachelys* is also cited as a TSD species [115], but the original source of this statement is lacking. Because of skewed sex-ratio in breeding programs, TSD is also very likely in *Geochelone platynota* [129] and *Pyxis* species [130]. No data exists for the genera *Chersina*, *Chersobius*, *Homopus*, *Indotestudo*, *Kinixys*, *Psammobates* or *Stigmochelys*.

**Figure 6:**
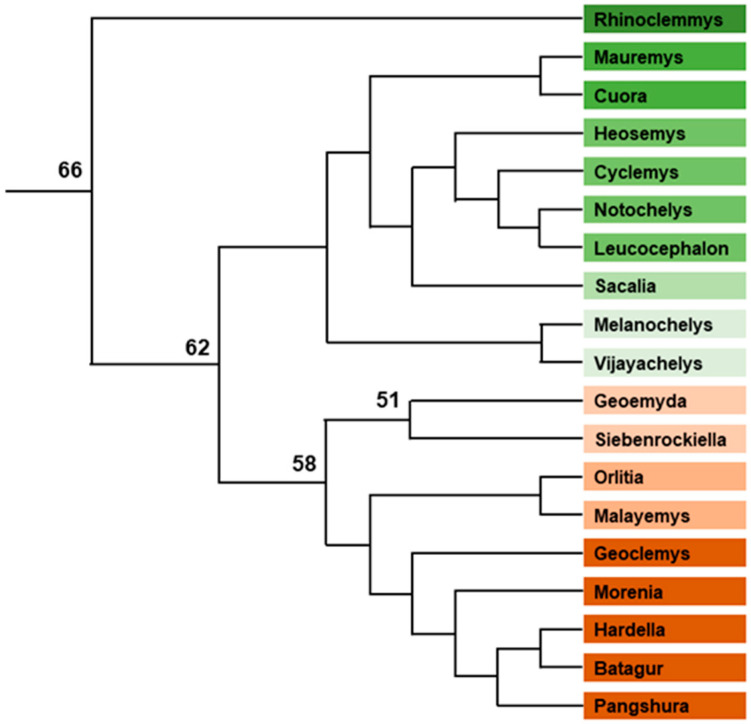
Phylogenic tree of geoemydid turtles. Relative position of each genus and superior clade are represented. Branch lengths are not proportional to time. Numbers indicate divergence time (Myrs) from the present for some key nodes, according to [39]. Color of each genus indicates the different clades.

Geoemydidae includes two subfamilies: Rhinoclemmydinae with 9 South or Central American *Rhinoclemmys* species, and Geoemydinae with 63 mainly South-Asian species composing 18 genera (Figure 6). TSD is documented in *Rhinoclemmys areolata* [48,73], *Rhinoclemmys pulcherrima* [48], *Mauremys annamansis* [73], *Mauremys nigricans* [73], *Mauremys mutica* [48,131,132,133], *Mauremys sinensis* [132], *Mauremys japonica* [134], *Mauremys reevesii* [135], *Cuora flavomarginata* [136], *Melanochelys trijuga* [48,73], *Heosemys grandis* [137] and *Malayemys macrocephala* [138]. Almost all studied species possess homomorphic chromosomes [89,139,140,141,142,143] with mostly 2n = 52, except in *Rhinoclemmys punctularia* (2n = 56) [142] and in the two sister genera *Orlitia* and *Malayemys* (2n = 50) [89,140,144]. A ZZ/ZW system has been described in *Pangshura smithii* [145], but this observation was later demonstrated to be an error [146]. The only known exception is *Siebenrockiella crassicollis* (2n = 50) which exhibits a heteromorphic pair of chromosomes, the X being submetacentric and the Y being metacentric [147,148]. *P. sinensis* chromosome 5 probes paint both *S. crassicollis* sex chromosomes exclusively [148], demonstrating the strict homology between them. As *P. sinensis* chromosome 5 is homologous to chicken chromosome 5 [149], *S. crassicollis* sex chromosomes are also homologous to chicken chromosome 5. Fourteen *S. crassicollis* homologs of chicken chromosome 5 genes were cloned, and all mapped to *S. crassicollis* X and Y chromosomes, demonstrating their overall synteny despite a different gene order on chicken and *S. crassicollis* chromosomes [148]. However, the gene order is conserved between *S. crassicollis* X and Y chromosomes, suggesting the absence of major rearrangements. This may be interpreted as the sign that sex chromosomes are at an early stage of differentiation in this species. Although all fourteen genes are localized on the long arm of the submetacentric X chromosome, the shift of the centromere put two genes on the short arm of the metacentric Y chromosome [148]. Among these fourteen genes, Wilms’ tumour 1 (*WT1*), which is located near the pericentromeric region of the Y chromosome where it begins to differentiate from the X chromosome, is especially interesting. This gene encodes a transcription factor containing four zinc finger motif DNA-binding domains, which play an important role in the early formation of the urogenital system and in gonads differentiation in vertebrates. Numerous isoforms of the protein exist, and a variation of particular interest involves the omission or insertion of three amino acids (KTS) between the last two zinc finger domains, giving −KTS or +KTS isoforms. In mice, the absence of +KTS isoforms leads to complete XY sex reversal [150]. Conversely, mice with a specific change in the fourth zinc finger mimicking a mutation found in 46XX humans with testicular disorders of sex development, show masculinization of the embryonic XX gonad [151]. The role of WT1 in turtle gonad differentiation has not yet been entirely elucidated, but in *Chelydra serpentina* the ratio of +KTS/−KTS isoforms is higher in developing gonads at male temperature than in those at female temperature [152]. Thus, *WT1* seems to be a good candidate gene for the role of master sex gene in *S. crassicollis*. However, neither X- or Y-specific *WT1* sequences or isoforms, nor differential levels of transcription from the X or Y *WT1* gene copy, have been described, and further study is needed before definitive conclusions can be reached. 

The genus *Siebenrockiella* consists of two species. One of these, *S. leytensis*, is one of the rarest and most endangered turtles in the world. As *Heosemys leytensis*, it was first erroneously described from the Philippine Island of Leyte [153] and years later rediscovered in Palawan Island [154] and transferred to the genus *Siebenrockiella* [155]. The two *Siebenrockiella* species diverged 30 million years ago [39]. Unfortunately, the karyotype of *S. leytensis* is still unknown and thus we still do not know its chromosomic number and if heteromorphic sex chromosomes occur in this species. The genus *Geoemyda*, which contains two species *G. spengleri* and *G. japonica*, is the sister group of the genus *Siebenrockiella* and diverged from it 50 million years ago [39]. Both species possess homomorphic chromosomes (2n = 52) similar to those of other Geoemydidae, and CGH fails to detect any sex-differences [146]. Their sex determination system (TSD or GSD) is also unknown. Given the apparently early state of sex chromosome differentiation in *S. crassicollis*, the homomorphic chromosomes in *Geoemydea* and the long-time divergence between the two genera, it is likely that genetic sex-determination in *S. crassicollis* is restricted to the genus *Siebenrockiella* or even to the sole species *S. crassicollis*. 

**Figure 7:**
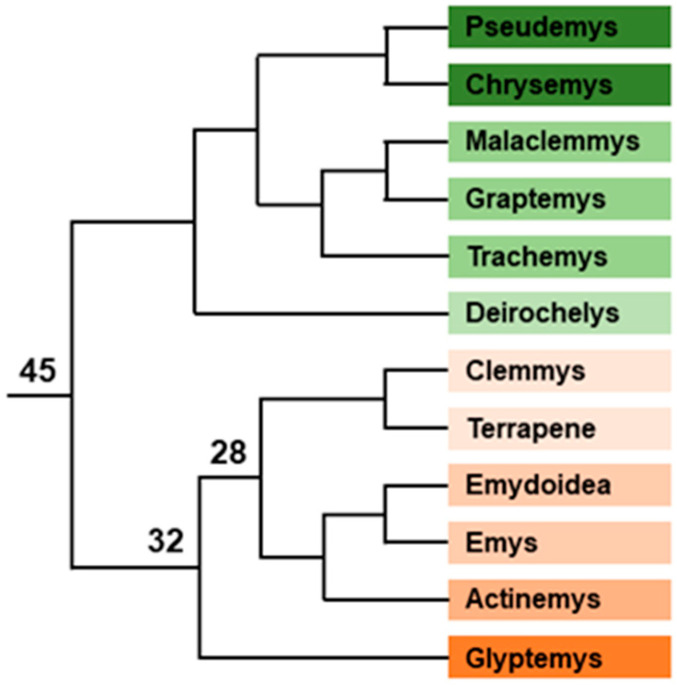
Phylogenic tree of emydid turtles. Relative position of each genus and superior clade are represented. Branch lengths are not proportional to time. Numbers indicate divergence time (Myrs) from present for some key nodes, according to [39]. Color of each genus indicates the different clades.

Emysternia is divided into two families, Platysternidae with the sole species *Platysternon megacephalum* (2n = 54) and Emydidae (2n = 50) with 12 genera and 51 species (Figure 7). Being mostly composed of North American species, Emydidae has been intensively studied and TSD has been documented in 20 species that belong to 11 of the 12 genera [30,48,73,137]. In the last genus *Glyptemys* (2 species), GSD was demonstrated for the species *G. insculpta* [74]. The karyotype of *G. insculpta* was first claimed to be devoid of heteromorphic chromosomes [95,156], but a reexamination with more modern methods has revealed the presence of a pair of heteromorphic chromosomes, the fourth largest in size, the X being subtelomeric and the Y being a little larger and submetacentic [157]. The process of chromosome Y differentiation is in a relatively advanced state with a different G-banding pattern compared to the X chromosome [157]. Moreover, the use of CGH shows the presence of three male-specific regions corresponding to the G-bands on the Y chromosome [157]. Lastly, hybridization with *C. picta* chromosome 4 BAC probes demonstrate the existence of two inversions between the X and Y chromosomes [157]. Thus, the Y chromosome has undergone many changes since it started to differentiate from the X chromosome. The sex chromosomes in *G. insculpta* are homologous to *C. picta* chromosome 4 [157], itself homologous to chicken chromosomes 5 (and 26) and *P. sinensis* chromosome 6 [158]. As *S. crassicollis* sex chromosomes are also homologous to chicken chromosome 5 and *P. sinensis* chromosome 6 [148], both *G. insculpta* and *S. crassicollis* sex chromosomes seem to be derived from the same part of the genome. It is tempting to speculate that the same region was selected twice by evolution because it encompasses an important gene of the sex differentiation network able to become a sex master gene driving evolution of a sex chromosome, but this remains to be proven. However, the *WT1* gene is localized on both *G. insculpta* X and Y chromosomes [91] and is a good candidate for such a role. The generation of molecular markers for sex diagnostic by PCR in *G. insculpta* has also led to the discovery of the same XY system of sex determination in the other species of the genus, *G. muhlenbergii* [159]. Consequently, this sex chromosome system arose after the split of the genus *Glyptemys* from its sister group containing the genera Terrapene, *Emys*, *Actinemys* and *Emydoidea* and before the split between the two *Glyptemys* species. In a paper describing the sex chromosomes in *G. insculpta*, the authors give −20 Myr and −8 Myr, respectively, for these events [157], while another paper gives a −32 Myr/−14 Myr window [39]. Whatever the true dates, the claim of *Glyptemys* sex chromosomes as the youngest sex chromosomes in turtles [157] is challenged by the poor degree of differentiation of the Y chromosome in *S. crassicollis* [148]. In this regard, the occurrence or not of sex chromosomes in *S. leytensis* will be of tremendous importance.

The last turtle family, the monotypic Platysternidae with the sole *P. megacephalum*, is the only turtle family with no information about its sex determination system. No incubation experiments at controlled temperature have even been performed. Only two publications give information about the *P. megacephalum* karyotype. One simply mentions that the karyotype is 2n = 54 and refers to a future publication about turtle karyotype before adding in the addendum that “the work cited as in preparation by Kiester and Childress has been discontinued, there are presently no plans for formal publication” [160]. The second presents the karyotype obtained from three males and one unsexed juvenile [141]. Therefore, we do not know if the karyotype of a female has ever been examined. Even the draft of the genome of this species was obtained from a male [161].

#### 2.1.3. General Considerations

Are there some possibilities that other sex chromosome systems exist in turtles? Some families such as Trionychidae, Kinosternidae or Emydidae have been highly investigated and offer no hope for this possibility, but some other taxa have been neglected and modern cytogenetics is lacking for them. The discovery of heteromorphic chromosomes in *G. insculpta* with modern cytogenetics is encouraging for efforts to reinvestigate some groups or species. For instance, the genus *Pelusios* in Pelomedusidae contains 15–20 species, but the karyotype is known for only four species and TSD has been demonstrated for only one species. South American Chelidae have been poorly investigated and merit a detailed study to determine if they share the same sex chromosome system as Australasian Chelidae. In Bataguridae, karyotype information is lacking for the genus *Morenia*, and the karyotype of some species such as *Geoclemys hamiltoni* is from unsexed animals [162]. Moreover, karyotypes of other species, such as *Orlitia borneensis* [142] or *Hardella thurjii* [89], are only known for one sex. In the genus *Siebenrockiella*, the karyotype of *S. leytensis* remains to be described and compared to those of *S. crassicollis*. 

**Figure 8:**
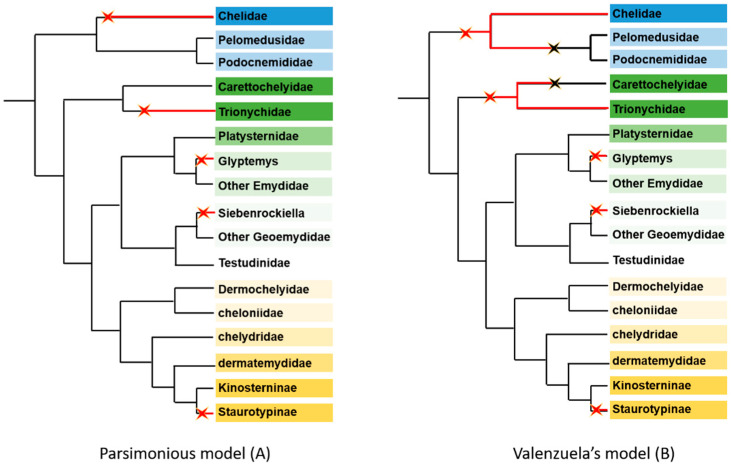
Models for transitions among sex-determining mechanisms in turtles. Black lines indicate branches where TSD occurs, red lines indicate branches where GSD occurs. Red stars indicate TSD to GSD transitions, black stars indicate GSD to TSD transitions. (**A**) Parsimonious model with five independent TSD to GSD transitions; (**B**) Valenzuela’s model with two older TSD to GSD transitions in pleurodira and trionychia, respectively, and two putative GSD to TSD transitions in pelomedusoides and carettochelyidae, respectively.

Overall, five different GSD systems exist in turtles. When looking at their phylogenetic distribution, the intuitive, simplest, and most parsimonious hypothesis is that these five systems arose independently from TSD (Figure 8A). Unexpectedly, the laboratory of N. Valenzuela published another model [50]. Using maximum likelihood procedures, they claimed that the transitions from TSD to GSD leading to GSD in Chelidae and Trionychidae were very old and occurred before they split from Pelomedusoides and Carettochelyidae, respectively. Accordingly, they postulated two putative transitions from GSD to TSD in both Carettochelyidae and the common ancestor of Pelomedusoides (Figure 8B). Moreover, the study of the rate of molecular evolution of genes implicated in sex-determination gave some support to this hypothesis [163]. If true, this model implies two testable consequences. Firstly, as recently pointed out by [164], it means that TSD in both Pelomedusoides and Carettochelyidae is a derived state and therefore not homologous to TSD in other turtles. Consequently, we expect a different mechanism for TSD in these families compared to other turtles. The deciphering of the mechanisms of TSD in reptiles has made tremendous advances recently [165,166,167]. In the turtle *Trachemys scripta elegans*, the key factor is Lysine Demethylase 6B (KDM6B or JMJD3), which promotes the male pathway by activating the male sex-determining gene *Dmrt1* [166]. At female-producing temperature, likely in response to a rise in Ca^2+^ levels, Stat3, Signal Transducer and Activator of Transcription 3, is phosphorylated and transcriptionally represses expression of *KDM6B*, pushing the gonad towards a female phenotype [167]. If such a mechanism were also active in Pelomedusoides and Carettochelyidae, this would not be in favor of Valenzuela’s model. Secondly, this model entails that, for example in Pelomedusoides, the autosome homologous to the X chromosome of Chelidae was an X chromosome during the interval between the two transitions of sex-determining mechanisms. Unlike autosomes, an X chromosome spends more time in females than in males. Because gametes experience fewer division cycles in ovary than in testis, mutations occur at a slower rate in females compared to males. Therefore, an X chromosome accumulates fewer mutations than an autosome, as documented in mammals [168]. So, if Valenzuela’s model is right, by comparing the substitution rate of X-linked Chelids genes with their autosomal orthologs both in Pelomedusoides and in an outgroup (some TSD cryptodirian species as *C. picta* for instance), one would expect a slower rate in Chelids and an intermediate rate in Pelomedusoides compared to outgroup species. The same type of reasoning can be made for the Z chromosome genes of Trionychidae and its orthologs in Carettochelyidae and a TSD Durocryptodirian outgroup, with the only difference being that the Z chromosome accumulates more mutations than autosomes. Of course, such analyses will require whole genome sequences of *Carettochelys* and Chelids, Pelomedusoides and Trionychids species, which are not yet available. However, with the growing number of available genomes, it will soon be possible to test Valuenzela’s model.

### 2.2. Lizards

**Figure 9:**
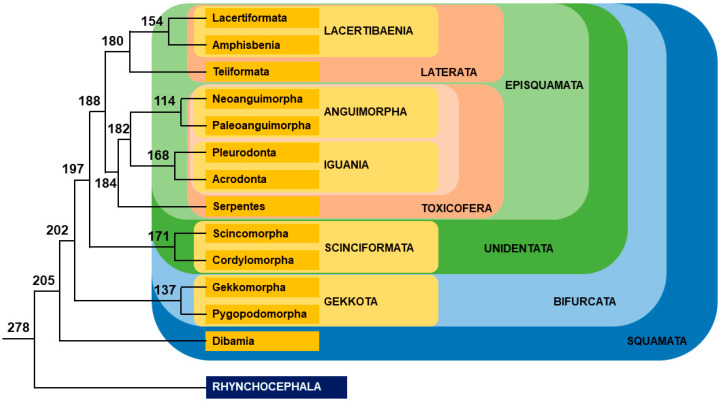
Phylogenic tree of squamates. Relative positions of each clade are represented following [38]. Names of clades are according to [170]. This tree has strong support, except for the position of Dibamia, which is sometimes alternatively placed as the sister group of Gekkota [171]. Branch lengths are not proportional to time. Numbers indicate divergence time (Myrs) from present for nodes, according to [38]. Colors indicate the different level of clades in the phylogenetic tree.

Among Lepidosauria, Rhynchocephala is represented by the two species of tuataras, which are TSD species [169]. All other Lepidosauria belong to Squamata, which is divided between the following groups (Figure 9).

#### 2.2.1. Dibamidae

Dibamidae is a small family of almost limbless fossorial vermiform lizards distributed in Mexico for the genus Anelytropsis (one species) and between South East Asia and New-Guinea for the genus Dibamus (24 species). Dibamidae is either the sister group of all other Squamata (Figure 9) [38] or the sister group of Gekkota, together forming the sister group of all other Squamata [171]. The karyotype of only one species, *Dibamus novaeguineae*, is known [172], and it shows the presence of a heteromorphic pair of chromosomes in males. This pair, the fourth largest, is composed of a submetacentric chromosome similar to the pair found in females and thus thought to be the X chromosome, and a telomeric chromosome specific to males and thought to be the Y chromosome. However, only one male and one female were studied, and this result needs confirmation. Whether XY chromosomes occur in other species of the family remains to be established.

#### 2.2.2. Gekkota

**Figure 10:**
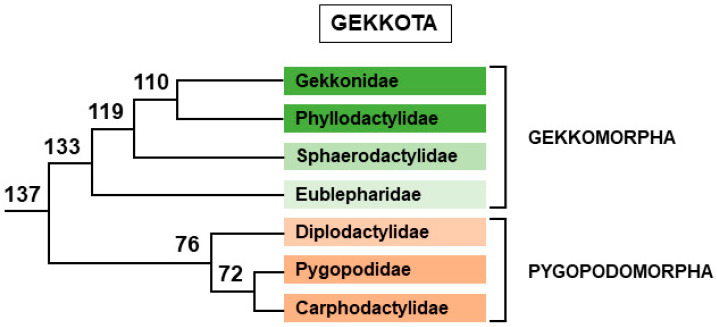
Phylogenic tree of Gekkota. Relative positions of each family are represented. Branch lengths are not proportional to time. Numbers indicate divergence time (Myrs) from present for nodes, according to [38]. Color of each family indicates the different clades.

With nearly 2000 species, Gekkota is one of the most numerous groups of lizards. Currently, it is divided into seven families but its phylogenetic tree is not yet stabilized [38,171], the position of the family Eublepharidae being the most contentious issue (Figure 10). Among Gekkota, the family Gekkonidae is by far the most numerous (1356 species). From early studies at the end of the twentieth century, it soon appeared that sex determination mechanisms were very diverse in this group. Some species, such as *Eublepharis macularius* possess TSD [173], whereas others exhibit sex chromosomes with either female heterogamy such as in *Christinus marmoratus* [174], or male heterogamy, such as in *Lialis burtonis* [175]. These transitions between sex determination systems can even occur in the same genus. For example, in the genus *Gekko*, *G. gecko* possesses an XX/XY sex chromosome system [176] and *G. houkouensis* a ZZ/ZW sex chromosome system [177]. With time, information about more species has accumulated and confirmed this variability [178,179,180,181]. In recent years, the use of restriction site-associated DNA sequencing (RAD-seq) helped greatly to identify sex determination systems in species with no known karyotype or homomorphic chromosomes. This technique amplifies and sequences DNA regions flanking specific restriction enzyme sites distributed throughout the genome in a number of male and female individuals. After validation, the presence of male specific markers indicates the species possesses XY chromosomes whereas female specific markers identify ZW chromosomes. In the case of TSD species, this experiment would give no sex specific fragment. This method requires only a few individuals of each sex (at least 5, ideally 10) for success. In Gekkota, RAD-seq experiments led to the identification of eight previously uncharacterized sex determination systems [182]. Coupled with a phylogenetic analysis, this revealed the existence of at least 15 independent transitions between sex determination systems in Gekkota, even within the same genus. For instance, in the genus *Hemidactylus*, *H. frenatus* has a ZZ/ZW sex chromosome system, whereas *H. mabouia* and *H. turcicus* possess an XX/XY sex chromosome system [182]. The RAD-seq method has succeessfully identified new sex chromosomes in a number of species or genera [183,184,185]. The lability of sex determination systems in Gekkota is reminiscent of what happens in frogs or some fishes, such as medaka [5,10,11,12,13,186,187].

Among all of these identified sex determination systems, only a handful have been studied in detail. In *Gekko hokouensis* from Okinawajima Island (Japan), the Z chromosome is acrocentric and the W chromosome subtelocentric [177]. Six homologues of chicken Z chromosome genes map to gecko Z and W chromosomes, demonstrating that they originated from the same pair of autosomes as in birds [177]. The differentiation of the sex chromosomes is rather advanced because several chromosome rearrangements must be postulated to explain the differences observed between the Z and W chromosomes. The *DMRT1* gene is present at different locations in the differentiated region of the Z and W chromosomes [177]. It represents a good candidate for the role of master sex-determining gene in this species, but there is no data about a difference in sequence or level of expression between the Z and W copies of *DMRT1*. Hybridization of *G. hokouensis* metaphases with Z and W chromosome probes of *Christinus marmoratus*, another Gekkonidae, marks the chromosome 5, not the sex chromosomes, demonstrating the sex chromosomes in these two ZW species are not homologous [188]. Moreover, just as *G. hokouensis* chromosome 5 is homologous to chicken chromosomes 23 and 4p [189], sex chromosomes of *C. marmoratus* are also homologous to chicken chromosomes 23 and 4p. Chicken chromosome 4p is homologous to mammals’ X chromosome, and contains at least two genes, *Sox3* and *AtrX*, which have been selected by evolution to become master sex-determining genes in other vertebrates. The only known member of the sex determination genetic network encoded by chicken chromosome 23 is the *Rspo1* gene. It is thus a possible candidate for the role of MSD gene in this species.

Another group investigated in detail is the genus *Paroedura* [180,190], another member of the Gekkonidae. By classic cytology, all studied species seem to possess homomorphic chromosomes, but C-banding and CGH revealed the presence of a heterochromatic W chromosome in females of most species [180]. However, in a small clade of species, nested within other species with differentiated sex chromosomes, these techniques fail to detect distinguishable sex chromosomes [180]. After identification in a species with a heterochromatic W chromosome, Z-specific markers show the presence of the same ZW system in all species with heterochromatic W chromosomes, and its absence in the clade without differentiated sex chromosomes [190]. Because this ZW system is also lacking in *Ebenavia*, the sister group of *Paroedura*, and other more distantly related geckos, it likely appeared in the last common ancestor of *Paroedura* geckos (62–90 Myr) and was later lost in the clade with no differentiated sex chromosomes. The sex-determination system in this latter clade is thus unknown. In the other *Paroedura* species, the Z-specific markers are homologs to genes located to chromosomes 4p and 15 in chicken [190]. As mentioned previously for *C. marmoratus*, chicken chromosome 4p has been co-opted several times to become sex chromosomes. Chicken chromosome 15 also shows homology with sex chromosomes of Trionychidae turtles and Iguana lizards (see below). In Gekkonidae, *Paroedura* is closer to *Christinus* than to *Gekko* [191], and both *Paroedura* and *Christinus* sex chromosomes share homology with chicken chromosome 4p. However, it is likely that there were independent co-option events in each genus and not common ancestry, because chicken 4p homolog genes are autosomal in other genera classified between *Paroedura* and *Christinus* [189].

Still in Gekkonidae family, a recent study revealed the existence of both ZZ/ZW and XX/XY sex chromosome systems in the genus *Cyrtodactylus* [192]. Female heterogamy had previously been described by classical cytology in the species *Cyrtodactylus pubisulcus* [193]. By RAD-seq experiments, hundreds of female-specific RAD markers were isolated from the species *C. pharbaungensis*, and two sex-specific PCR primer pairs were validated, demonstrating this species possesses a ZZ/ZW sex chromosome system [192]. However, the same approach with the species *C. chaunghanakwaensis* led to the isolation of 166 male-specific markers and the validation of three sex-specific PCR primer pairs, indicating an XX/XY sex chromosome system. Comparing these male specific markers against chicken genes using the basic local alignment search tool (BLAST) gave only two results on two different chicken chromosomes, and it was thus impossible to conclude about the homology of the *C. chaunghanakwaensis* sex chromosome in chicken [191]. With *C. pharbaungensis*, the BLAST comparison identified 38 chicken genes, half on them belonging to chicken chromosome 10 [192]. It is thus likely that the *C. pharbaungensis* sex chromosomes are syntenic to chicken chromosome 10. As no known member of the sex determination genetic network is located on chicken chromosome 10, no candidate for the role of MSD gene in *C. pharbaungensis* has been proposed so far. The sex-specific PCR primer pairs validated in *C. pharbaungensis* have not yet been tested in *C. pubisulcus*. Therefore, whether the two species share the same ZZ/ZW sex chromosome system or not is unknown.

In Phyllodactylidae (152 species), another gecko family, RAD-seq experiments allowed the discovery of a ZW sex chromosome system in the species *Phyllodactylus wirshingi* [184]. When compared with BLAST against chicken genes, only four of the 539 female-specific RAD contigs gave results, and all of them matched genes on the chicken Z chromosome. Thus, in *P. wirshingi*, sex chromosomes are homologs of chicken sex chromosomes [184]. 

In Sphaerodactylidae (229 species), a third gecko family, the same type of RAD-seq experiments demonstrated the occurrence of a ZZ/ZW system in four species of the genus *Aristelliger* [185]. Five out of 878 female-specific markers are homologs of genes located on both arms of chicken chromosome 2 (or for one of them, on chicken chromosome 33) which corresponds to *Anolis* chromosome 6 [194]. The linkage of these markers with sex chromosomes has not been tested in other genera close to *Aristelliger*, such as *Quedenfeldtia* or *Teratoscincus*. Therefore, the extent of this ZZ/ZW system among Sphaerodactylidae remains to be determined. Chicken chromosome 2 is also homologous to sex chromosomes of two different snake lineages (see below). No known member of the sex-determination genetic network is located on chicken chromosome 2, but the authors of the study highlight the presence of β-catenin (*CTNNB1*) and 5α-reductase (*SRD5A1*) genes on this chromosome [185].

In Eublepharidae (44 species), a recent study identified an X_1_X_1_X_2_X_2_/X_1_X_2_Y sex chromosome system in *Coelonyx elegans* by comparing genome coverage between sexes [194]. The identified X-specific genes have homologs on chicken chromosomes 1, 6 and 11 [194]. The extent of this sex chromosome system among other Eublepharidae geckos was investigated by qPCR experiments. Only *C. mitratus*, the closest relative of *C. elegans* [195,196], shares the same system as *C. elegans* [194]. More precisely, both *Coelonyx variegatus* and *C. brevis*, which are also GSD species [197] but with homomorphic chromosomes [198], lack this sex-determination system. Their sex-determination system is thus still unknown. As *C. mitratus* and *C. elegans* diverged from other *Coelonyx* species around 34 Myrs ago, their sex-determination system is at least as old.

In Carphodactylidae (32 species), female heterogamy was first demonstrated in the species *Underwooddisaurus milii* by molecular cytogenetic methods [179]. More recently, whole genome sequencing from *Saltuarius cornutus*, another member of the family Carphodactylidae, and *U. milii* was performed [199]. Comparative genome coverage analysis between sexes led to the discovery of Z-specific genes in both species. These Z-specific genes were then tested in other species of the family by qPCR experiments. In *S. cornutus* and three other species of the genus *Saltuarius*, Z-specific genes are orthologs of genes from chicken chromosomes 17, 22 and 24, whereas in *U. milii* and five species of the genus *Nephrurus*, they are orthologs of genes from chicken chromosome 10, and to a lesser extent from chicken chromosome 17 [199]. It is not known if there are two different ZZ/ZW systems in the genus *Saltarius* on one hand and the genera *Nephrurus* and *Underwooddisaurus* on the other hand, or if all these species share a common sex chromosome system with an MSD gene homologous to a chicken chromosome 17 gene. In the case of two independent ZZ/ZW systems, they are at least 15–36 Myr old in *Nephrurus* and *Underwooddisaurus*, and 16 Myr old in *Saltarius*. If there is a unique sex determination system, it is at least 29–46 Myr old [199]. Both regions homologous to chicken chromosome 10 and 17 have been co-opted several times in lizards (*C. pharbaungensis*, *Pogona*, Corytophanidae) to become sex chromosomes.

Lastly, in Pygopodidae, a small family of Australasian limbless geckos (46 species in 6 genera), the species *L. burtonis* was studied in detail [200]. Similar to *L. jicari*, the other species of the genus, *L. burtonis* exhibits X_1_X_1_X_2_X_2_/X_1_X_2_Y sex chromosomes [181], whereas two other Pygopodidae species, *Aprasia parapulchella* and *Delma butleri*, possess XX/XY sex chromosomes [201]. Transcripts from *L. burtonis* blood cells (two females and four males) and from one male *L. jicari* were sequenced, and the sequences were identified by comparison with chicken and other reptilian genomes. Genes with no SNPs in all five males were selected, and their chicken homologs mapped to the chicken genome. These genes were scattered with roughly the same density over all chicken chromosomes, except on the long arm of chicken chromosome 4 (4q) which is exceptionally enriched in such genes, suggesting this syntenic block is the homolog of the X-specific region of *L. burtonis* [200]. This hypothesis was confirmed by qPCR experiments, which demonstrated that genes homologous to chicken chromosome 4q genes are X-specific not only in *L. burtonis* but also in *L. jicari* and three other Pygopodidae genera distributed throughout the Pygopodidae family. As this XY chromosome system is shared between four different genera, it is likely it arose before the differentiation of genera from the last common ancestor of Pygopodidae, at least 30 Myrs ago [200]. However, it is restricted to Pygopodidae, as female heterogamy occurs in its sister group, Carphodactylidae (Figure 10) [179,199], and so posterior to the divergence between the two families some 55 to 78 Myrs ago [200]. Moreover, the analysis of gene expression in male and female blood cells shows that only genes homologous to chicken chromosome 4q genes differ in their expression between sexes, these genes being significantly less transcribed in males than in female [200]. In other words, there is no global gene dosage compensation in *L. burtonis*.

To summarize, chromosomic regions homologous to chicken chromosomes 2, 4p, 10, 17 and Z have been regularly co-opted independently in several groups of geckos and other reptilian lineages, but some gecko lineages have co-opted other unique chromosomic regions homologous to chicken chromosomes 1, 4q, 6, 11, 22 and 24. As recently emphasized by [199] these changes of sex chromosome systems in Gekkota are different than those observed in some fishes or amphibians because they are much more older and evolutionary stable [199].

#### 2.2.3. Scinciformata

**Figure 11:**
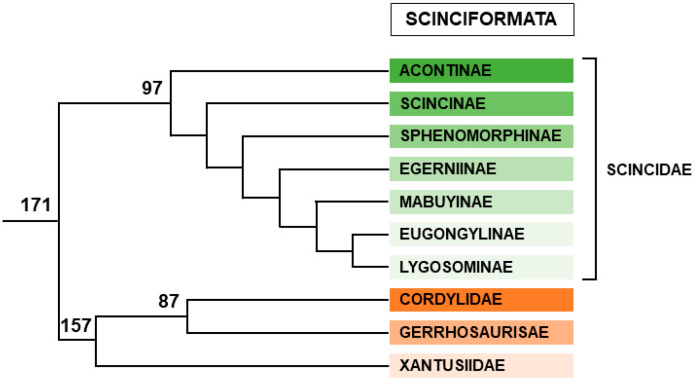
Phylogenic tree of Scinciformata. Relative positions of each family or subfamily are represented. Branch lengths are not proportional to time. Numbers indicate divergence time (Myrs) from present for nodes, according to [38]. Color of each family or subfamily indicates the different clades.

This phylum is divided into Scincomorpha on one side, with the huge family Scincidae (1715 species), and Cordylomorpha on the other side, with three small families: Cordylidae (70 species), Gerrhosauridae (37 species) and Xantusiidae (35 species) (Figure 11). In Cordylidae, all karyotyped species possess homomorphic chromosomes, and their sex determination system is unknown. In Gerrhosauridae, conventional cytogenetic methods revealed homomorphic chromosomes in all studied species, but a ZZ/ZW sex chromosome system was recently suggested in the species *Tracheloptychus petersi* [202]. In this species, CGH fails to reveal sex-specific part of a chromosome, but an rRNA probe marks two minichromosomes in males and only one in females [202]. Taken together, these results argue in favor of poorly differentiated sex minichromosomes in *T. petersi* [202]. Heteromorphic chromosomes are also unknown in Xantusidae, but a study identified a ZZ/ZW sex chromosome system in *Xantusia henshawi* [203]. This RAD-seq experiment recovered no male-specific and 267 female-specific RAD markers, demonstrating the presence of ZZ/ZW sex chromosomes. When compared against chicken and anole lizard genomes, these female-specific RAD markers matched with 4 and 16 genes, respectively. Of these genes, four were located on chicken chromosomes 7, 12 and 18, eight of them on anole chromosome 2 (homologous to chicken chromosomes 12 and 18), and the remaining eight on unmapped scaffolds or anole chromosomes 1 and 3 [203]. Attempts to use these markers on two other *Xantusia* species were unsuccessful, and therefore the extent of this ZZ/ZW sex chromosome system across Xantusiidae is still unknown. However, another recent study showed that parthenogenic females of *Lepidophyma smithii*, another Xantusiidae species, could yield offspring of both sexes, suggesting that female is the heterogametic sex in this species as well [204]. *Lepidophyma* is the sister group of *Xantusia*, and it is therefore possible that the two genera share the same ZZ/ZW system, but this remains to be proved. The authors of the *Xantusia* study note the presence of *Sox9*, a major component of the male pathway in vertebrates and direct target of the male-determinant factor SRY in mammals, on chicken chromosome 18 and likely on anole chromosome 2. However, they did not mention that *CBX2*, another well-known member of the genetic sex-determination network, is also located on chicken chromosome 18 and on anole chromosome 2. CBX2, a polycomb protein, plays a major role in sex-determination because it seems to be a powerful repressor of normal ovarian development in mammals. In mice, the disruption of its gene leads to male to female sex reversal in homozygous mutants [205]. In human, a normal girl with a 46XY karyotype was found bearing one different missense mutation of *CBX2* on each allele of the gene [206]. Moreover, in two rodent species of the genus *Tokudaia* where both sexes are X0 with no *SRY* gene, *CBX2* is present in multiple copies in both sexes and there are two or three more copies of *CBX2* gene in males than in females [207]. Therefore, *CBX2* is a strong candidate for the role of master sex-determining gene in *Tokudaia*. It would be thus interesting to assess the possible roles of both *Sox9* and *CBX2* in *Xantusia* sex determination.

Skinks constitute a very successful (1718 species) cosmopolitan family since almost one of every four species of lizards belongs to their family Scincidae. Scincidae contains seven subfamilies, the most basal being Acontinae, and a second split separates Scincinae from the five remaining subfamilies (Figure 11). As with many lizards, most skinks possess homomorphic chromosomes, and heteromorphic XY chromosomes occur in only around 25 species scattered all over the family, with the highest concentration in some Australian skinks in which the heteromorphic pair is the seventh largest. Concerning sex determination, the situation has long been puzzling and the family was best known for some oddities. This included viviparous species with TSD in the genera *Eulamprus* [208] and *Niveoscincus* [209], a TSD species with heteromorphic sex chromosomes, *Bassiana duperreyi* [210], and a species, *Niveoscincus ocellatus*, with TSD-like and GSD in lowland and highland populations, respectively [211,212]. Further research has demonstrated sex-reversal by temperature and existence of Y-chromosome markers in *B. duperreyi* [213,214], and conservation of sex-linked markers in both populations of *N. ocellatus* [215]. Therefore, these two species likely are GSD species in which temperature can override GSD. Two recent studies shed light on a shared conserved XX/XY sex chromosome system in most skinks [216,217] In the first study, the authors sequenced male and female genomes of *Scincus scincus* (from basal subfamily Scincinae) and compared the coverage of exons between sexes [217] They identified 560 genes with half coverage in males compared to females. Then, they filtered these genes for the absence of polymorphisms in males, before mapping them to the high-quality genome of *Podarcis muralis*, another lizard belonging to Lacertidae. Among the 169 genes they recovered, 37 localized to a 7 Mb region on the tenth chromosome of *P. muralis* [217]. Using qPCR, they tested 10 of these X-linked genes among 13 skink species covering all the subfamilies with the exception of Acontinae and demonstrated that all the species shared common X-linked markers. In contrast, these genes are autosomal in the three other Scinciformata families (Cordylidae, Gerrhosauridae and Xantusiidae), indicating that this XX/XY chromosome system is restricted to Scincidae and originated between 150 and 85 Myr ago. The skink X-linked genes have homologs in chicken chromosome 1 [217]. This region contains several candidate genes for the role of master sex-determining genes, such as *Stra8*, *Sox10*, *ep300* and *Sbf1*. A second study utilized a quite different transcriptional approach in the species *Eulamprus heatwolei* [216]. After obtaining transcriptomic data from brain, liver and gonads of males and females, a subtraction approach allowed the identification of Y-linked transcripts of 14 protein-coding genes. These 14 gametologs are all orthologs of genes located on a single block located on chicken chromosome 1 and anole chromosome 5 [216]. One of these gametologs is *UBE2H*, which was also identified as a Y-specific marker in *B. duperreyi* [218]. Moreover, five of the Y-linked markers previously identified in *N. ocellatus* [215] map to the same region of anole chromosome 5, indicating these two skinks share a homologous Y-chromosome [216]. From synonymous substitution rates of the gametologs and the divergence time between these two skinks, the authors estimate the sex chromosomes originated between 116 and 80 Myr ago [216]. This is in good agreement with the minus 150–minus 85 Myr interval estimate of the other study [217]. Among the 14 identified gametologs, the authors emphasize *PPP1R12A* (Protein Phosphatase 1 Regulatory subunit12A), a gene that encodes an enzyme that interacts with the phosphoprotein phosphatase 1 catalytic subunit (PPP1C), the major dephosphorylation complex in cell. In humans, mutations in the *PPP1R12A* gene can lead to urogenital anomalies including sex-reversal in XY individuals [219]. Moreover, the *PPP1CC* gene, one of the members of the PPP1C complex, is a good candidate for the role of master sex-determining gene in Pleurodonts, another reptile lineage (see below), and the knock-out of its testis-specific isoform in mice leads to male sterility [220]. So, several results point to a role of proteins of the PPP1C complex in sex-determination. Unexpectedly, a ZZ/ZW chromosome system with differentiated chromosomes was discovered in *Scincella melanostica*, an Asian member of the genus [221]. This was very surprising because heteromorphic XY chromosomes have been described in *S. lateralis*, a North American member of the genus, and are also suspected to exist in two other North American *Scincella* species [222,223]. Differentiated XX/XY and ZZ/ZW chromosomes in the same genus are quite unusual in reptiles, the other known examples being in the gecko genera *Gekko*, *Hemidactylus* and *Cyrtodactylus* [182,192]. It should be very interesting to investigate the genus *Scincella* in detail using the Y-specific markers identified in other skinks.

Thus, Scincidae, with some exceptions in the genus *Scincella*, share a common ancestral XX/XY chromosome system with a genomic sex-specific region homologous to chicken chromosome 1 and anole chromosome 5, whereas a ZZ/ZW chromosome system with a genomic sex-specific region homologous to chicken chromosomes 7, 12 and 18 and anole chromosome 2 occurs in *Xantusia* and likely other Xantusiidae. In Gerrhosauridae, *T. petersi* possesses a ZZ/ZW chromosome system too, but its homology with the *Xantusia* system or lack thereof is unknown.

#### 2.2.4. Laterata

**Figure 12:**
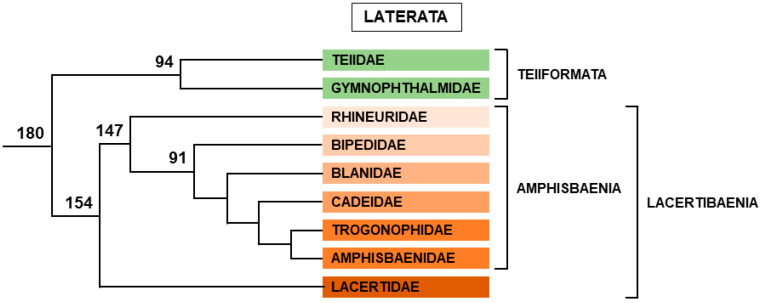
Phylogenic tree of Laterata. Relative positions of each family are represented. Branch lengths are not proportional to time. Numbers indicate divergence time (Myrs) from present for nodes, according to [38]. Color of each family or subfamily indicates the different clades.

Laterata are divided between Teiiformata with two families, Teiidae (171 species) and Gymnophthalmidae (270 species), and Lacertibaenia, composed of Amphisbenia (6 families, 203 species) on one side, and a single family, Lacertidae (354 species) on the other side (Figure 12).

In Teiidae, all species studied so far show homomorphic chromosomes, except *Cnemidophorus tigris* [224] and *C. littoralis* [225] which have conspicuous XY chromosomes. Whether this XY chromosome system extends to other genera or the whole family is unknown. In Gymnophthalmidae, heteromorphic XY chromosomes are less rare but restricted to only seven species of the sub-family Gymnophthalminae, in the genera *Micrablepharus*, *Nothobachia*, *Calyptommalus* and *Gymnophthalmus* [226,227,228,229]. All other tested species in subfamilies Gymnophthalminae, Ecpleopinae and Cercosaurinae possess homomorphic chromosomes. Whether this XY chromosome system occurs in the whole family and is homologous with those observed in the sister group Teiidae is not known.

Amphisbenia is a small group of fossorial limbless worm-like lizards, composed of 5 small families and the more numerous family Amphisbaenidae (182 species). Only karyological information is available for this group. One species, *Bipes tridactylus*, has a heteromorphic fourth pair of chromosomes in female and thus a ZZ/ZW system [230]. This result was established from the study of two males and three females and seems robust. The two other members of the family Bipedidae, *Bipes biporus* and *Bipes canaliculatus* [230]), and all other species studied so far possess homomorphic chromosomes. Examination of the whole group by modern methods will help to assess the extent of a ZZ/ZW system in Amphisbenia.

Lastly, Lacertidae (354 species), the common European lizards, includes species with homomorphic chromosomes and more than 35 species with female heterogamy (see Table 1 in [231], for complete references). The group of L. Kratochvíl and M. Rovatsos first identified Z-linked genes by looking for female transcripts devoid of SNPs in the species *Takydromus sexlineatus* [232]. Among the 85 genes found, most (51) had chicken orthologs scattered on 14 different chromosomes, but 23 genes had orthologs on chicken chromosome 4p and 11 genes had orthologs on chicken chromosome 17. After qPCR validation, they established that the Z-specific region of *T. sexlineatus* is homologous to chicken chromosome 4p, itself homologous to marsupial chromosome X, the X-conserved region of human chromosome X, and chicken chromosome 17 [232]. qPCR experiments later extended this result to *Lacerta agilis* and 43 other species from 25 genera covering the entire phylogenetic range of Lacertidae [231,232]. Therefore, all Lacertidae share the same ZW system, even if one or two genes sometimes exhibit autosomal (or pseudoautosomal) behaviour in one or two species, reflecting a species-specific variation of the pseudoautosomal region or species-specific translocation of some genes on autosomes. Moreover, these Z-linked genes are autosomal in Teiidae and Blanidae (an amphisbaenian family), suggesting that this ZW sex determining system took place after the split between Lacertidae and Amphisbaenia around 150 Myrs ago, and before the split between the two sub-families of Lacertidae, approximatively 85 Myrs ago [233]. However, it would be very interesting to test these lacertid Z-linked markers in *B. tridactylus*, the only amphisbaenian with proven ZW chromosomes.

The only known gene of the sex determination genetic network localised on chicken chromosome 17 is *NR5A1* (Nuclear receptor subfamily 5, Group A, Member 1) (=*Sf1*, Steroidgenic factor-1). In humans, its mutation has been associated with XY sex reversal [233], and it has a very important role in testis differentiation in mice [234]. More candidates lie on chicken chromosome 4p, especially *Sox3*, *AtrX* and *AR* (androgen receptor). Two paralogs of *Sox3*, *SRY* in mammals and *Sox3Y* in the fish *O. dancena* [5], have acquired the role of master sex-determining gene. In the axolotl *A. mexicanum*, *ATRW* the master sex-determining gene, having originated from a duplication of *ATRX* [27]. Lastly, *AR* is suspected to be the male sex-determinant in the ZW type of *Rana rugosa* frogs [235]. It must be noted that at least one other unknown candidate exists in this syntenic block. Four species of rodents independently evolved a special feminizing X chromosome, noted X*, able to override the effect of SRY and leading to the existence of X*Y female (see [236] for review). It is tempting to speculate that it is the same unknown gene which was selected and mutated to assume this role in the four species. This unknown gene is perhaps specific to mammals or even rodents, but it could also be an important conserved gene of the sex-determination network among vertebrates. If so, it could also be a good candidate for the role of master sex-determining gene in Lacertidae. Whatever the master sex-determining gene in Lacertidae, its mode of action is still unknown. In lizards from the genus *Darevskia*, ZZZW tetraploids are male and ZZW triploids are mostly females but with some males or intersexual animals [237]. This is rather in favour of a dosage of Z-linked gene mechanism than of a W-dominant gene mechanism, but there is still no definitive evidence.

To summarize our knowledge of GSD in Laterata, Teiiformata possess one or several XY systems, whereas only ZW systems exist in Lacertabaenia. All lacertids share the same ZW system, which arose from the co-option of a genomic block homologous to chicken chromosomes 4q and 17. This lacertid ZW system is not present in at least one Amphisbaenian family, Blanidae (7 species), but it is still unknown if it is homologous or not with the ZW system of *B. tridactylus*, another Amphisbaenian.

#### 2.2.5. Toxicofera

**Figure 13:**
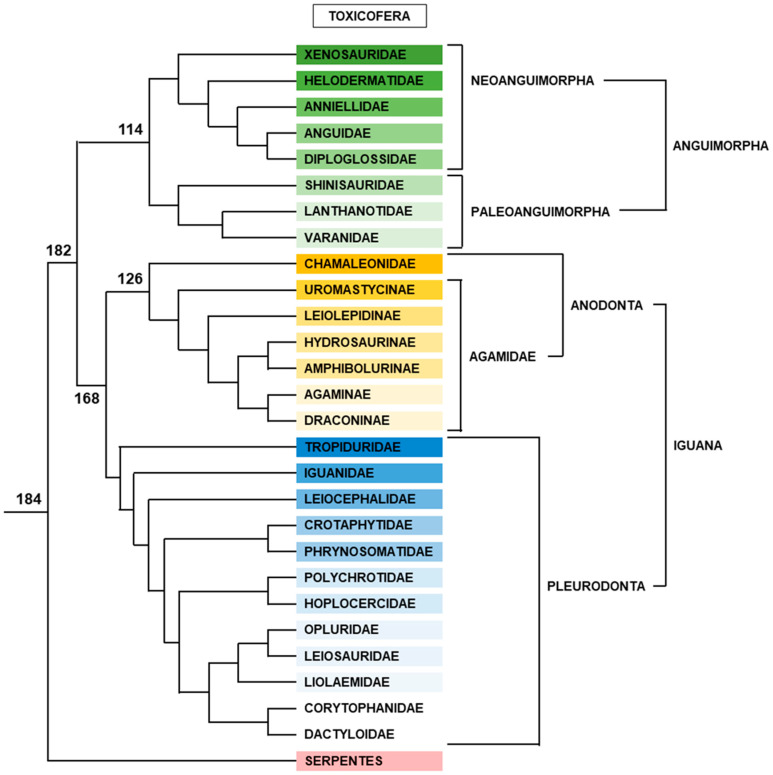
Phylogenic tree of Toxicofera. Relative positions of each family and superior clade are represented. Branch lengths are not proportional to time. Numbers indicate divergence time (Myrs) from present for nodes, according to [38]. Color of each family or subfamily indicates the different clades.

This clade is the most numerous one (more than 6000 species) among Squamates. It is divided between snakes (Serpentes) in one side, and two groups of lizards (Anguimorpha and Iguana) on the other side (Figure 13).

##### Anguimorpha

This group includes Paleoanguimorpha, with the family Varanidae (84 species of monitors (genus *Varanus*)) and two monotypic families (Lanthanotidae and Shinisauridae), and Neoanguimorpha, divided in Xenosauridae (12 species), Helodermatidae (5 species), Anniellidae (6 species), Diploglossidae (51 species) and Anguidae (81 species) (Figure 13).

The first evidence of sex chromosomes came from the study of the genus *Varanus* with the description of a ZZ/ZW sex minichromosomes in four species [238,239]. Later, this ZW system was discovered in other species [240,241,242]. Lastly, chromosome painting probes of *Varanus komodoensis* allowed the discovery of ZZ/ZW sex minichromosomes in six other species and demonstrated that the Z chromosome was conserved in 12 species covering all the phylogenetic diversity of the genus [243]. Therefore, all monitors share the same ZW system. 

In another family, Helodermatidae, C-banding and CGH identified Z and W chromosomes in the Gila monster, *Heloderma suspectum* [244]. This raised the question of the homology of ZW systems between Varanidae and Helodermatidae. To answer this question, L. Kratochvíl and M. Rovatsos followed the same strategy that was used for Lacertidae and identified Z-linked genes by looking for female transcripts devoid of SNPs in several *Varanus* species [245]. They identified a Z-specific region in *Varanus* as homologous to chicken chromosome 28 and validated this result by qPCR experiments. Moreover, by testing Z-specific primers for *apba3* and *grin3b* genes in different species, they determined that these genes were Z-specific in 21 *Varanus* species, three *Heloderma* species and *Abronia lythrochila*, an Anguidae species [245]. Therefore, this ZW system is older than the divergence between Paleoanguimorpha (Varanidae) and Neoanguimorpha (Helodermatidae and Anguidae), around 115 Myr. As these genes are autosomal in Iguana and Serpentes species, this ZW system is specific to Anguimorpha. The occurrence of this ZW system in other Anguimorpha families (Diploglossidae, Xenosauridae, Anniellidae, Lanthanotidae and Shinisauridae) is unknown. Interestingly, in another Anguidae species, *Anguis fragilis*, these two genes are autosomal or pseudoautosomal [245]. This suggests either a change of sex chromosomes or more likely a longer pseudoautosomal region in this species. Another very important result of this study is the lack of dosage compensation in the expression of Z-linked genes in blood cells of Komodo dragon, *V. komodoensis* [245]. In a later study, the occurrence of a ZW system of sex chromosomes was confirmed or suggested by cytogenetics in more species. More precisely, in *A. lythrochila* a putative W chromosome was identified on a minichromosome by C-banding, and both C-banding and CGH revealed a female specific signal in two other Anguidae species: *Celestus warreni* and *Gerrhonotus liocephalus* [246].

Chicken chromosome 28 contains one major gene of the genetic sex-determination network: the *Amh* (anti-Müllerian hormone) gene. This gene has been independently co-opted several times to become the master sex-determining gene in some fishes, such as *O. hatcheri* [15] and the pike *E. lucius* [16]. It is also suspected to play this role in several other (but not all) *Odontesthes* species [247] and other fishes [248,249]. Lastly, it is very likely that *Amh* has also been recruited to become the MSD gene in monotremes, which exhibit the very unusual X_1_X_2_X_3_X_4_X_5_/Y_1_Y_2_Y_3_Y_4_Y_5_ sex chromosome system. In Platypus, it exists as two copies, namely *AmhY* located on sex chromosome Y_5_, and *AmhX*, located in the oldest strata S0 (because the synonymous nucleotide substitution rate is highest between X_1_-Y_5_ gametologs) of sex chromosome X_1_ [21,22]. Therefore, *Amh* has been suggested as a very good candidate for the role of MSD gene in Anguimorpha [245].

##### Iguana

Iguana is divided in two clades: Anodonta and Pleurodonta (Figure 9 and Figure 13).

Anodonta

This group includes two families, Chamaeleonidae (chameleons) and Agamidae (dragon lizards) (Figure 13). Sex-determination and sex chromosomes are rather poorly known in Chamaeleonidae (217 species). Old dubious records of TSD in genus *Chamaeleo* (see in [250]) were later demonstrated to be erroneous [197,251]. Controlled-temperature incubation experiments confirmed GSD in chameleons [197,252], but classical cytology failed to detect heteromorphic sex chromosomes in most species. However, CGH and C-banding experiments revealed the existence of female heterogamety in the genus *Furcifer* [253]. More specifically, *F. oustaleti* showed a classical ZW system with a highly heterochromatic W chromosome, whereas *F. pardalis* possessed a quite unusual Z_1_Z_1_Z_2_Z_2_/Z_1_Z_2_W multiple sex chromosome system. This neo sex chromosome system likely arose from the classical one through a W-autosome fusion [253]. Later, this rare Z_1_Z_1_Z_2_Z_2_/Z_1_Z_2_W multiple sex chromosomes system was recovered in three other *Furcifer* species, and a classical ZZ/ZW system in *F. lateralis* [254]. However, CGH experiments failed to detect heteromorphic sex chromosomes in other genera such as *Calumma*, *Chamaeleo*, *Rieppeleon* and *Trioceros* [255]. The study of *Rhampholeon temporalis* by C-banding indicates this species may possess a ZZ/ZW system because heterochromatic blocks are different in the two chromosomes of the 8th pair in females; however, no males were examined and therefore this result needs confirmation [255]. *Chamaeleo calyptratus* is among the species where CGH failed to detect heteromorphic sex chromosomes [255]. However, RAD-seq experiments identified 13 male-specific and 2 female-specific RAD markers in this species, suggesting an XX/XY sex chromosome system [256]. Moreover, five PCR primer pairs, derived from the male-specific markers, confirmed this result [256]. Four of these five PCR primer pairs amplified specific bands in male only in the close species *Chamaeleo chamaeleon*, demonstrating the two species share the same XX/XY sex chromosome system [251]. FISH experiments with the amplified fragment of one of these PCR primers pairs as a probe specifically stained the pericentromeric region of the short arm of only one of the two chromosomes 2 in male, identifying it as the Y chromosome [251]. Chromosome painting with chicken Z chromosome probe had previously identified the short arm of *C. calyptratus* chromosome 2 as the homolog of chicken Z chromosome [257]. It is thus possible that the sex chromosome of the two *Chamaeleo* species is homologous to the chicken Z chromosome, but this requires further investigation. As for the chicken homolog of *Furcifer* chromosome Z, results have not been published yet but in [258], the authors cited chicken chromosome 4p as a personal communication. In conclusion, Chamaeleonidae are GSD species with poorly differentiated homomorphic sex chromosomes, except in genus *Furcifer*, which possesses heteromorphic sex chromosomes, and at least one ZW system and one XY system exist in the genera *Furcifer* and *Chamaeleo*, respectively. In other genera, the extent of these systems or the occurrence of other systems remain to be investigated.

The second family, Agamidae (534 species), is very important because TSD was discovered in one of its members, *Agama agama* [29], and because this family exhibits the greatest variety of sex-determination in lizards, including TSD species, GSD species and GSD species where temperature can override genetic sex determination (see later). In Agamidae, heteromorphic sex chromosomes are rather rare and TSD seems to be the rule, but our level of knowledge is quite variable for the different subfamilies. In subfamilies Uromastycinae, Leiolepidinae and Hydrosaurinae, only some karyotypes are known, and they show no sign of heteromorphism, but their sex determination systems have never been determined. In the subfamily Draconinae, known karyotypes exhibit homomorphic chromosomes, and only the species *Calotes versicolor* has been studied in some detail. This species was first claimed to be a GSD species because of a lack of effect of temperature on sex-ratio [259], but later on, other experiments demonstrated TSD with a quite unusual FMFM pattern [260] and failed to identify sex-specific markers with RAD-seq [261]. Therefore, *C. versicolor* is now considered a TSD species [262]. In the subfamily Agaminae, TSD was demonstrated in several *Agama* species and ZW heteromorphic chromosomes were observed only in *Phrynocephalus vlangalii*, whereas other species of this genus possess homomorphic chromosomes [263]. This species is the only member of the family to have sex macrochromosomes. Nothing is known about the gene content of these sex macrochromosome pairs and their homology with chicken chromosomes, but a high-quality male genome and transcriptome of both female and male organs of *P. vlangalii* are available [264,265]. Therefore, the identification of gametologs by a transcription subtraction approach, such as those developed by D. Cortez laboratory could be possible. The last subfamily, Amphibolurinae, is the best studied. Its species are mostly Australian or New-Guinean, with only two reaching south-eastern Asia. Both TSD and GSD exist in this subfamily, and sometimes even in the same genus. For instance, the genus *Amphibolurus* contains both the TSD species *A. muricatus* and the GSD species *A. norrisi* ([250]. So far, GSD is known in the genera *Amphibolurus*, *Ctenophorus*, *Diporiphora*, *Hypsilurus*, *Pogona*, *Rankinia*, and *Tympanocryptis* [250,266]. However, sex chromosomes have been described in only 5 species (*Pogona vitticeps*, *Pogona barbata*, *Diporiphora nobbi*, *Ctenophorus fordi* and *Tympanocryptis lineata*), and in all cases they are ZZ/ZW sex minichromosomes [262,267,268]. Agamid ZZ/ZW sex minichromosomes were first discovered in *P. vitticeps* by CGH and C-banding [267]. It became the model species to study sex determination in this group. After isolation of markers of its Z and W sex chromosomes, establishment of its molecular cytogenetic map, and lastly sequencing of its genome, it was determined that its sex chromosomes are mostly homologous to chicken chromosome 17 and to a lesser extent homologous to chicken chromosome 23 [268,269,270,271,272]. The total sequence assigned to Z chromosome is at least 8.34 Mbp with 219 genes [272]. The MSD gene is still unknown, but *Rspo1*, the only known member of the sex determination genetic network encoded by chicken chromosome 23, has been ruled out because it is localized on an autosome in *P. vitticeps* [273]. Among genes homologous to chicken chromosome 17 genes, *NR5A1* is a strong candidate because it was mapped to *P. vitticeps* sex chromosomes by BAC hybridization, and because mutations in human and its knock-out in mouse both lead to male-to-female sex reversal [233,274]. Two other species, *P. barbata* and *D. nobbi*, share the same ZW sex determination system as *P. vitticeps* because the *P. vitticeps* sex-specific probe PvZW3 marks their sex minichromosomes [268]. Conversely, the same probe labels another minichromosome pair than the sex minichromosomes in *C. fordi*, suggesting a different ZW sex determination system than that observed in *P. vitticeps* [268]. As *Tympanocryptis* is relatively close to *Pogona* and *Diporiphora*, it is possible that *T. lineata* shares the same ZW sex determination system as *P. vitticeps*, but this remains to be demonstrated.

The influence of temperature is another very interesting point about sex-determination in *P. vitticeps*. Incubation of *P. vitticeps* eggs between 22 °C and 32 °C produced the expected 1:1 sex-ratio, but between 34 °C and 37 °C there was an increasing female bias leading to an almost 100% female production at higher temperatures, which could not be explained by differential lethality [275]. Moreover, genotyping with a W-specific probe demonstrated that at higher temperatures, half of the phenotypic females are genotypic males (ZZ). Thus, high temperature can override genotypic sex determination in in *P. vitticeps* [275]. This phenomenon is not restricted to laboratory experiments because up to 20% of females collected in the wild turned out to be ZZ sex-reversed females [276]. These ZZ sex-reversed females were fully fertile, and when mated with normal ZZ males gave birth to viable ZZ offspring the sex of which was determined by temperature only [276]. Thus, in only one generation, a GSD species can become a TSD species. This finding obliged researchers to reconsider the previous prevailing idea that GSD to TSD transitions are difficult and rare, and that sex chromosomes are an evolutionary trap [277]. However, it must be noted that despite the presence of numerous ZZ sex-reversed females in the wild, no isolated natural population of entirely ZZ individuals of both sexes has been discovered yet, suggesting there is an equilibrium between GSD and TSD that prevents the population from becoming fully TSD. The fact that the *P. vitticeps* W chromosome is not mandatory to produce females argues in favor of a male Z-gene dosage model of sex determination rather than a female-dominant W-gene model.

Pleurodonta

This species-rich group (1223 species) includes 12 families (Figure 13), the most numerous being the family Dactyloidae (436 species). Classical cytogenetics has revealed that most species show no sign of chromosomal heteromorphism. However, the first lizard heteromorphic sex chromosomes were discovered in this clade, more precisely in some species of the genera *Scleroporus* and *Anolis* [278,279]. Only male heteromorphism was known in Pleurodonts. Among the species with homomorphic sex chromosomes, the species *Anolis carolinensis* from the family Dactyloidae became an important model organism and was the first non-avian reptile to have its genome sequenced [280]. In this species, FISH experiments with BAC probes led to the identification of the X chromosome as a minichromosome homologous to chicken chromosome 15 [280]. In this first study, at least 5.1 Mb of sequence, containing 62 protein-coding genes, was assigned to this X chromosome, named Linkage Group b (LGb) [280]. However, numerous scaffolds remain unanchored in the assembly, and it was hypothesized that some of them could belong to the X chromosome. By looking into unanchored scaffolds containing genes homologous to chicken chromosome 15 and testing them by qPCR in male and female genomic DNA, the number of X-linked genes increased to 250 [281]. Later, a comparative analysis between chicken and anole genomes suggested there were at least 374 genes on the anole X chromosome [282], but another study restricted this number to 313 [283]. The FISH and qPCR experiments also demonstrated that the Y chromosome was highly differentiated and lacking most of the X-chromosome genes. 

The identification of the X chromosome gene content allowed testing of the homology of sex chromosomes among Pleurodonts. Chromosome painting with sex chromosome paints, sequencing of chromosome-specific DNA, FISH of X-linked BACs, and qPCR of X-linked genes demonstrated that all *Anolis* species, even those with large heteromorphic XY chromosomes or the multiple chromosome system X_1_X_1_X_2_X_2_/X_1_X_2_Y, share the same X chromosome gene content and thus the same sex-determination system [284,285,286,287]. Moreover, qPCR experiments with female and male genomic DNA led to the discovery of homologous sex chromosomes in almost all other pleurodont families [285,288,289], with the exception of the family Corytophanidae [288,289]. Therefore, this sex chromosome system arose before the basal split between the different families 73–93 Myrs ago, and likely after the split between Pleurodonta and Acrodonta 123–168 Myrs ago. However, this last statement has been challenged by the study of gametologs.

The first attempt to identify the Y-chromosome gene content used RAD-seq experiments and led to the discovery of the only *RTDR1Y* gene and its X-linked partner *RTDR1* (Rhabdoid tumor deletion region gene 1) [290]. A more successful male-female transcriptome/genome subtraction approach identified the complete coding sequences of seven protein-coding genes, including the previously known *RTDR1Y* gene [283]. Three of them, *RPL6Y* (Ribosomal protein L6), *UBE2L3Y* (Ubiquitin-conjugating enzyme E2L3) and *EWSR1Y* (Ewing sarcoma breakpoint region 1), exhibited ubiquitous expression like their X-counterparts and homologs in other species. *RTDR1Y* was gonad specific similar to *RTDR1* on the X chromosome and in other species. The last three genes, *PPP1CCY* (Protein phosphatase 1, catalytic subunit gamma isozyme), *DENRY* (Density-regulated protein) and *SLC5A1Y* (Solute carrier family 5 (sodium/glucose cotransporter), member 1) had acquired testis-specific expression, whereas their X-counterparts and homologs in other species were ubiquitously expressed (*PPP1CC* and *DENRY*) or possessed kidney-specific expression (*SLC5A1Y*) [283]. Moreover, phylogenetic trees of ancient XY gametologs and outgroup orthologs based on synonymous site divergences revealed that *RPL6* and *PPP1CC* belong to the oldest stratum S1 and that they likely both originated before the split between Pleurodonta and Acrodonta 149–172 Myrs ago. The other Y-linked genes evolved after this split. Taken together, these results indicate that *PPP1CCY* is a very strong candidate for the role of MSD gene in Pleurodonts [283]. The *PPP1CC* gene is a member of the PPP1C (phosphoprotein phosphatase 1 catalytic subunit) complex, the major dephosphorylation complex in cells, and the knock-out of its testis-specific isoform in mice leads to male sterility [220]. Moreover, *PPP1R12A* (Protein Phosphatase 1 Regulatory subunit12A), a gene that encodes another enzyme interacting with the PPP1C complex, is a good candidate for the role of MSD gene in skinks, and in humans its mutation can lead to urogenital anomalies including sex-reversal in XY individuals [219]. Therefore, more and more, the role of the PPP1C complex and its components in sex-determination appears worthy of further investigation.

The origin of the pleurodonts XY sex-determination system before the split between pleurodonts and anodonts implies that this system persisted for a certain time in the ancestors of anodonts before the appearance and evolution of the ZW sex-determination systems observed today. A study addressed this question by analyzing male mutation bias in pleurodonts, anodonts and snakes [291]. During gametogenesis, male gametes undergo more replication cycles than female gametes, hence a greater mutation rate due to replication errors. Male mutation bias is the name given to this phenomenon. If we assume a 1:1 sex ratio in a population of an XY species, autosomes spend half their time in males and half their time in females, whereas the Y chromosome spends all of its time in males and the X chromosome spends one third of its time in males and two thirds in females. Consequently, the Y chromosome is expected to evolve faster (i.e., accumulate more mutations) than autosomes, whereas the X chromosome is expected to evolve more slowly than autosomes. The comparison between the synonymous substitution rates of pleurodonts X-linked genes and those of their autosomal homologs in agamids and snakes shows that, among the tested species, the synonymous substitution rate is the highest in the six snakes because their genes remained autosomous since the split between snakes and Iguanans around 184 Myrs ago, the lowest in the three pleurodonts because their genes stayed X-linked since the appearance of the pleurodonts’ XY system (149–172 Myrs ago) just after the split between snakes and Iguanans, and intermediate in the three agamids because their genes had remained X-linked for several million years before becoming autosomous during agamid evolution [291]. Of course, this difference between the three groups is specific for pleurodonts’ X-linked genes and was not observed for pleurodonts’ autosomal genes. Interestingly, among the three agamid species, *P. vlangalii*, a ZW species from the subfamily Agaminae, showed a clearly higher synonymous substitution rate than *P. vitticeps* and *Ctenophorus decresii*, two species from the subfamily Amphibolurinae with ZW chromosomes or TSD, respectively [291]. This means that the common ancestor of Amphibolurinae species retained the pleurodont XY system longer than *Phrynocephalus vlangalii*. In other words, in Agamids, the *Pogona* ZW system is independent from and younger than the *Phrynocephalus vlangalii* ZW system. An estimation of the divergence times indicates that *Phrynocephalus vlangalii* lost the pleurodont XY system 105 Myrs ago, whereas the *Pogona/Ctenophorus* clade lost it only 20–60 Myrs ago [291].

The appearance of a dosage compensation mechanism in *A. carolinensis* constitutes another major discovery brought by the study of pleurodonts [282,283]. An early study, based on analysis of regenerating tail transcriptomes of different animals and determination of the male/female expression ratio for each gene, found a slightly incomplete dosage compensation (0.87 ratio, versus 1.01 ratio for autosomal genes) of X chromosome genes in this species [282]. More specifically, it was suggested that complete dosage compensation occurred for the genes annotated on LGb (0.97 ratio) and incomplete dosage compensation (0.82 ratio) for those located on other scaffolds assigned to the X chromosome [282]. A more complete study analysed transcriptomes of four tissues and both gonads in *A. caroliniensis*, chicken, and four mammals [283]. Expression level of X-linked genes in anole was found to be the same in each sex, and similar to ancestral expression levels estimated from the expression levels of autosomal homologs in other studied species. Moreover, a male specific two-fold X chromosome expression up-regulation restoring the ancestral expression level was demonstrated [283]. This male-specific up-regulation is mediated by a male-specific change in the chromatin machinery. This is achieved by a specific elevated level of H4K16ac (H4 histone acetylated on the lysine 16) on the X chromosome in males, whereas autosomal H4K16ac levels were similar in males and females [283]. Interestingly, the expression of the *APBB1* gene was the second most male-biased in anole lizard. Its protein, APBB1, is a cofactor of a histone acetyl transferase from the MYST superfamily called KAT5 which is able to up-regulate gene expression by acetylation of histones of the nucleosome. It is thus tempting to hypothesize that APBB1 is involved in the dosage compensation mechanism in *A. caroliniensis*. This complete dosage compensation in anole lizard shares many similarities with the dosage compensation mechanism in the fly *Drosophila melanogaster*. In this species, male specific X chromosome up-regulation also occurs through acetylation of H4K16ac on the X chromosome following the action of MOF, another histone acetyl transferase from the MYST superfamily (see [292], for review). This is a striking example of convergent evolution in two highly divergent groups.

Lastly, the family Corytophanidae possesses a different XY chromosomes system. A first study used RAD-seq experiments to identify male-specific RAD tags in *Basilicus vitttatus* [293]. These male-specific RAD markers revealed synteny between the *B. vittatus* sex chromosome chicken chromosome 17, and this synteny was confirmed by qPCR of genes homologous to chicken chromosome 17 genes. These qPCR experiments in other species of the family confirmed that they all share the same synteny with chicken chromosome 17, but also that some genes behaved similar to autosomal or pseudoautosomal in some species, suggesting a variation in length of the pseudoautosomal region, a translocation of some genes on autosomes, or a lack of differentiation of the gametologs on the X and Y chromosomes [293]. The authors noted the presence of the *NR5A1* gene on chicken chromosome 17, and dated the appearance of this new XY system between 15–50 Myrs. Lastly, the comparison between male and female eye transcriptomes revealed a lack of dosage compensation in *B. vittatus* [293]. Another study used a quite different genomic and transcriptomic approach [294]. The sequencing of the *B. vitttatus* genome showed that the read coverage of sequences orthologous to the *A. carolinensis* X chromosome were the same in males and females, and that homologs of the *A. carolinensis* Y chromosome were no longer present in the genome of *B. vittatus* [294]. Thus, the general pleurodont XY system had disappeared from *B. vittatus* genome. The transcriptomic subtraction approach in *B. vittatus* led to the identification of 12 protein-coding Y-specific genes, all orthologous to genes located on chicken chromosome 17. Only 4.5% of chicken chromosome 17 genes had orthologs on the *B. vittatus* Y chromosome, illustrating the highly differentiated state of *B. vittatus* sex chromosomes [294]. Among the twelve Y chromosome gametologs identified, only two, *EHMT1Y* and *ZBTB34Y*, showed regulatory functions, and only five, including *EHMT1Y*, were also present in the genome of *Corytophanes hernandesii*, another corytophanid [294]. Therefore, *EHMT1Y*, a histone lysine methyltransferase 1 encoding gene, seemed the most promising candidate for the role of MSD gene in corytophanids. However, it was expressed in many somatic tissues similar to its ancestor and was not testis specific as expected for an MSD gene. Most other Y-linked gametologs showed unusual features such as low-level expression, ubiquitous expression, or gain of kidney or blood cell expression compared with their ancestors [294]. A possible explanation for this lack of Y gametologs with the expected features of a “good” MSD gene is that the MSD gene might not be a Y chromosome gene, but rather a dosage sensitive X chromosome gene. If this were the case, the study of other corytophanid species with less differentiated sex-chromosomes and longer pseudo-autosomal regions could be very helpful [193]. Contrary to the first study, male and female transcriptomic data from four somatic tissues and gonads indicated a partial dosage compensation [294]. This could be a problem for the dosage sensitive X chromosome gene hypothesis, except if the dosage took place after the sex determination of the gonad. Whatever the MSD gene is, the study of gametologs and calibration by fossils dated the origination of this new XY system around 63 Myrs [294].

The ancestral pleurodont XY system is thus an old system (149–172 Myrs) which was present in the common ancestor of all species of the Iguana clade. It has persisted in Pleurodonts except in corytophanids where it was lost around 63 Myrs ago and replaced by a new XY system with sex chromosomes homologous to chicken chromosome 17. It was also lost in all Anodonts, apparently several times independently, as its disappearance was dated to 105 Myrs ago for *Phrynocephalus vlangalii* and 20–60 Myrs ago for the *Pogona/Ctenophorus* clade. Furthermore, the new ZW system which replaced it in this latter clade also possesses sex chromosomes homologous to chicken chromosome 17. Many questions remain. Why was such a system so stable in some lineages and lost several times in other lineages? When was it lost in Chamaeleonidae and in other Agamidae, especially in the TSD species of Agaminae? Is there a reason for the independent co-option of chromosomes homologous to chicken chromosome 17 to replace the ancestral pleurodont XY system? Could this ancestral pleurodont XY system be traced back before the divergence between Anguimorpha and Iguana? Only through whole genome comparisons and detailed analyses could we begin to answer these questions.

A more detailed description of lizard karyotype features, sex determination and sex chromosome systems can be found in the present Special Issue “Sex chromosome evolution and Meiosis” [295].

### 2.3. Snakes

**Figure 14:**
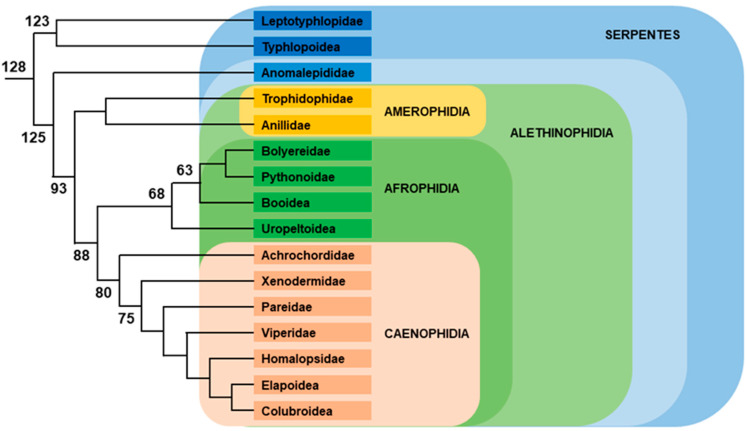
Phylogenic tree of snakes. Relative positions of each family and superior clade are represented. Branch lengths are not proportional to time. Numbers indicate divergence time (Myrs) from present for nodes, according to [38]. Colors indicate the different level of clades in the phylogenetic tree.

From a phylogenetic point of view, snakes are nested among lizards in the Iguana clade. Their phylogeny is complex and was only resolved by molecular studies (Figure 14) [38,171,191]. Among Squamates, they constitute a very successful group (3921 species). Most snakes belong to the clade Caenophidia (or modern snakes) (3238 species) which is the most recent clade to appear. The well-known boas and pythons and related families form the sister group of Caenophidia among Afrophidia (Figure 14). Snakes played an important role in the elaboration of the model of sex chromosomes evolution. By comparing chromosomes from different snake families, Susumu Ohno observed that boas possess homomorphic chromosomes whereas both Colubridae and Viperidae (two Caenophidian families) have heteromorphic ZW chromosomes. He also noted that Z and W chromosomes are of equal size and differ only by a pericentric inversion in most Colubridae, whereas the W chromosome is highly degenerated and smaller than the Z chromosome in Viperidae [296]. These observations led him to propose that in vertebrates, sex chromosomes evolve from an autosome pair, first are homomorphic, then become heteromorphic but still of equal size, and finally become highly divergent in size [297].

#### 2.3.1. Caenophidia

All snakes from this group studied so far exhibit ZW sex chromosomes, even those from the two most basal families: Acrochordidae [298] and Xenodermidae [299]. The first snake cytogenetic map, constructed by BAC FISH experiments in the species *Elaphe quadrivirgata* (Colubridae), revealed that the snake Z chromosome is mostly homologous to chicken chromosome 2 and for a smaller part to chicken chromosome 27 [300]. Only three of the eleven probes marking the Z chromosome hybridize with the W chromosome too [300]. Moreover, these eleven probes are also localized to the Z chromosome of the species *Prothobothrops flavoviridis* (under the older name *Trimeresurus flavoviridis*) (Viperidae), but none of them mapped to the W chromosome, suggesting a more differentiated state of the W chromosome in this species [300]. These probes map to Anole lizard chromosome 6 [301]. Genome sequencing of *Thamnophis elegans* (Colubridae) and *Sistrusrus miliarus* (Viperidae) confirm these results [301]. Analysis of genomic coverage of the scaffolds in both males and females revealed that in both species scaffolds homologous to *A. carolinensis* chromosome 6 (ACA6) show a nearly 2-fold reduction in female coverage compared to male. These scaffolds are therefore part of the Z chromosome, whereas the coverage is the same for scaffolds homologous to other Anole lizard macrochromosomes. This is not the case for the non-caenophidian snake *Boa constrictor* (Boidae), which exhibited similar male and female coverage for all macrochromosomes [301]. Further analyses revealed the presence of 712 putative Z-linked genes and 61 W-linked genes in *S. miliarus*, and 723 putative Z-linked genes and 29 W-linked genes in *T. elegans* [301]. Another important result is the presence of at least two and more likely three evolutionary strata on Z chromosomes of both species, the older strata being central in the chromosome. Lastly, comparison of Z-linked genes expression in both sexes of *B. constrictor* and *S. miliarus* versus *A. carolinensis* demonstrate the absence of global dosage compensation in snakes [301]. The occurrence of a Z chromosome homologous to ACA6 in other caenophidian families was tested by qPCR experiments using six pairs of ACA6 gene specific primers [302]. This demonstrated that the Z chromosome is homologous to ACA6 in all caenophidian snake families (Acrochordidae, Xenodermatidae, Pareatidae, Viperidae, Homalopsidae, Colubridae, Elapidae, Lamprophidae). Only one gene, tanc2, seems autosomal or (more likely) pseudoautosomal in the most basal family, Acrochordidae [302]. Interestingly, all genes homologous to ACA6 genes are autosomal not only in Toxicofera lizards but also in non-caenophidian snakes from the families Pythonidae, Xenopeltidae, Boidae, Erycidae and Sanziniidae which belong to the sister group of Caenophidia [302]. Therefore, all caenophidian snakes share the same ZW sex chromosome system, which appeared in their last common ancestor. So far, there is no candidate gene for the role of MSD gene in Caenophidia, even if some important genes for gonad development or sex determination, such as Dazl or CTNNB1 (β-catenin), localize on ACA6. Even if the MSD gene is still unknown, the important observation of a male *Elaphe bimaculata* (Colubridae) with ZZW sex chromosomes shed light on the mode of sex determination in caenophidian snakes [303]. This is more in favour of a dosage of a male gene on the Z chromosome, such as in birds, than in favour of a female master gene on the W chromosome. If this scenario is indeed the case in Caenophidia, the MSD gene should not be searched for in the gametologs of the most degenerated W chromosomes (Viperidae, Elapidae), but rather in the species with the least differentiated sex chromosomes, perhaps in the Acrochordidae family.

#### 2.3.2. Other Snakes

Ohno’s model assumed that boas, and by extension other related families, share the same ZW sex chromosome system with Caenophidia. With time, several lines of evidence cast some doubt on this statement. First, sequencing of *B. constrictor* genome fails to detect sequence difference between males and females in the macrochromosome homologous to ACA6 [301]), and all genes homologous to ACA6 tested so far are autosomal in boas and pythons [302]. Second, facultative parthenogenesis yields only male offspring in caenophidian snakes which is the expected result in a ZW species, but only female offspring in boas and pythons, a result expected for an XY species, and not easy to explain for a ZW species [304]. Lastly, a linkage study of a colour mutation called “coral glow” (CG) in the ball python (*Python regius*) demonstrated it was a sex-linked incomplete dominant mutation whose inheritance is not consistent with a ZW species, but rather with an XY species [305].Because both males and females can be phenotypically identified as homozygous or heterozygous for this sex-linked mutation, CG is likely localized in the pseudoautosomal region of the sex chromosomes. Moreover, as no other known traits are sex-linked in this species, the authors hypothesized that the sex chromosomes could be a pair of minichromosomes in *P. regius* [305]. They also hypothesized there is a possibility “that the henophidian sex chromosome share homology with the X and Y sex chromosomes of the pleurodont iguanians”. Finally, they concluded that “identifying the molecular underpinnings of the henophidian sex determination system will require further investigation [e.g., identification of sex specific markers via RAD-seq]” [305]. Such RAD-seq experiments were published by another group one year later [306]. In their study these authors looked for the presence of an excess of female or male-specific markers in a boa (*Boa imperator*), a python (*Python bivittatus*) and a rattlesnake (*Crotalus atrox*), used as a caenophidian snake control [306]. As expected for a ZW species, they found an excess of female-specific RAD markers for the rattlesnake. However, they found an excess of male-specific RAD markers for boa and python, confirmed this result by PCR validation, and therefore demonstrated the presence of XY chromosomes in both species. Interestingly, the boa male-specific RAD marker confirmed by PCR is also male-specific in the very closely related species *B. constrictor*. On the contrary, the primers of the python male-specific RAD markers confirmed by PCR failed to amplify in a sex-specific manner in two related species: the ball python (*P. regius*) and the carpet python (*Morelia spilota*) [306]. The extent of this XY system among other python species is therefore unknown. The mapping of sex-specific RAD-seq markers to the boa genome and the search for sex-specific SNPs led to the identification of sex-specific scaffolds, most of them bearing genes homologous to the Anolis linkage group f (LGf) (i.e., one of the *Anolis* microchromosomes). Among the two python PCR-validated male-specific markers, only one gave a BLAST result in the python genome, which identified a scaffold homologous to Anolis chromosome 6 (ACA6). Moreover, among the five identified python transcripts with male-specific SNPs, three of them mapped to genomic regions homologous to ACA6 [306]. So, it seems that the same chromosome, homologous to ACA6, was independently selected to become a sex chromosome in *P. bivittatus* and caenophidian snakes, but as part of an XY and ZW system, respectively, whereas another chromosome, homologous to Anolis LGf was recruited in boas. This variability in sex chromosomes in snakes was also illustrated by the recent discovery of heteromorphic ZW sex chromosomes in *Acrantophis sp. cf. dumerili*, one of the four or five species in the family Sanziniidae [307]. This pair of heteromorphic sex chromosomes remains an exception in boas and pythons, as CGH and other cytogenetic studies failed to detect sex-specific differences in ten other species from the families Calabariidae, Sanziniidae, Candoiidae, Charanidae, Boidae and Pythonidae [307,308]. Lastly, C-banding identified a heterochromatic minichromosome in a female *Myriopholis macrorhyncha*, a scolecophidian snake, suggesting the presence of a ZW system of sex chromosomes in this species [309]. However, a unique female was examined, and thus this result needs to be confirmed with more animals of both sexes.

To summarize, although all caenophidian snakes share the same ZW sex chromosome system homologous to Anolis chromosome 6, non-caenophidian snakes exhibit variation in their sex chromosome systems, with at least two different XY systems identified in boas and pythons, and one ZW system identified in the boa *A. sp. cf. dumerili*.

## 3. The Lessons from the Study of Sex Chromosomes in Reptiles

### 3.1. What We Learned

During the last two decades incredible progress has been accomplished in the knowledge of sex chromosomes in reptiles, and it has profoundly changed our vision about sex determination in reptiles on many points. The first point is the relative abundance of TSD and GSD in reptiles. In turtles GSD is relatively rare (94 GSD species versus 262 TSD species), but it is now clear that GSD is the rule in Squamates and TSD rarer than previously thought. Currently, TSD is clearly documented in two groups only (Gekkota and Agamidae). Even in Gekkota, its occurrence is demonstrated in only a few genera (*Eublepharis* and *Hemitheconyx* in Eublepharidae, *Tarentola* in Phyllodactylidae, *Phelsuma* in Gekkonidae) (see [199] for references). Temperature can overrule GSD in a few skinks (*Bassania*, *Niveoscincus*) or agamids (*Pogona*), but it is rather rare. Moreover, phylogenetic reconstitution of sex-determination mechanisms among Squamates shows many transitions from TSD to GSD, or from GSD to another GSD (named turnover), but transitions from GSD to TSD seem restricted to the Agamidae family. In reptiles, the general evolution trend is thus from TSD to GSD, and a return to TSD likely constitutes a rare event.

The discovery of so many GSD systems in reptiles has changed the scientific community’s opinion about the nature of these GSD systems. The discovery that many reptile groups (Chelidae, Tryonichidae, Scincidae, Pleurodonts, Lacertidae, caenophidian snakes) exhibit stable sex-chromosomes, as old as those of birds or mammals, was unexpected. Even geckos, previously thought to present a high lability of sex-determination systems, possess long-term stable sex-chromosomes that are more than 20 Myrs old [199]. Consequently, reptiles are similar to other amniotes in their stability of sex-determination systems, and different from amphibians and fishes, in which rapid turnover of sex-determination systems may occur, leading to very young sex chromosomes [13,186,187]. Nevertheless, some groups (geckos, non-caenophidian snakes, chameleons) show different sex-determination systems. Unsurprisingly, non-caenophidian snakes do not share the same sex determination system because they are very diverse, and their different clades diverged from each other million years ago. For instance, boas and pythons diverged from each other just after their divergence from caenophidian snakes, and the basal split between different families of blind snakes is much older. Our knowledge of sex determination systems in non-caenophidian snakes is too restricted to draw any conclusions but finding different sex-chromosome systems in their different families would not be unexpected. The same is true for geckos. As recently emphasized by [199], “the extant gekkotan families represent very old radiations, with the basal splits estimated to 57–180 Myrs.” Thus, the occurrence of different sex chromosome systems in geckos reflects the deep phylogenetic divergence among gecko genera or families. Relatively recent turnovers between sex-chromosome systems seem restricted to a few genera (*Scincella* in Scincidae, *Cyrtodactylus*, *Gekko* and *Hemidactylus* in Gekkonidae) or families (Sphaerodactylidae, Chamaeleonidae). Such changes in sex-chromosome systems are not a specific reptilian feature. Even in mammals, which are the prototype for long-term stable sex chromosome systems, unknown different sex determination systems exist in several rodent species (for instance *Nannomys minutoides*, *Ellobius lutescens* and *E. tancrei*, *Tokudaia osimensis* and *T. tokunoshimensis*) and two different sex determination systems can occur in the same genus (*Ellobius lutescens* versus *E. fuscocapillus*) [236]. Therefore, reptiles do not appear very different from other amniotes. 

Among amniotes, the only specific sex-chromosome change event in reptiles can be found in Agamidae family. In the subfamily Amphibolurinae, it is likely that the ancestral pleurodont’s XY system was lost 20–60 Myrs ago, replaced by TSD, and that later (around 25 Myrs ago) a new ZW system appeared in the *Pogona/Ctenophorus* clade [294]. If true, it would be the only known change from one sex-chromosome system to another one through an intermediate TSD system. The other possibility is a direct turnover from the ancestral pleurodonts XY system to the new *Pogona/Ctenophorus* ZW system in the last common ancestor of all species in Amphibolurinae, followed by a transition to TSD in some species. Whatever the exact scenario, a change from a sex-chromosome system to a TSD system, similar to those demonstrated in the laboratory for *P. vitticeps* [276], is mandatory to explain TSD species in Amphibolurinae. Such a change is the only known example in Amniotes. Other transitions between two GSD systems can be explained by different theoretical hypotheses such as genetic drift, sexually antagonistic selection, accumulation of deleterious mutations or selection on sex ratio (see [310,311] for review). The discussion of these hypotheses is beyond the scope of this review, but for most transitions in reptiles, such as those observed in Pleurodonts/Corytophanidae, genetic drift (i.e., the emergence of a new MSD gene on an autosome, strong enough to overrule the ancestral MSD gene and leading to the disappearance of the old Y or W chromosome) seems the simplest explanation.

Table S1 summarizes our current knowledge of reptilian clades with GSD and sex chromosomes, including homology with chicken (GGA) chromosomes when known. In theory, any autosome can evolve into a sex chromosome if it receives a translocated copy of a dominant sex-determining gene, such as *Dmrt1*. However, when looking at the homology of reptilian sex chromosomes with GGA chromosomes, some GGA chromosomes are present several times, suggesting they were repeatedly selected by evolution to become sex chromosomes. For instance, GGA 17 appeared five times, GGA Z and GGA 4p four times, and GGA 2 and GGA 15 three times. Some support for this non-random selection hypothesis was statistically found among amniotes [258]. The most likely explanation for the recurrent recruitment of the same genomic regions is the presence of important genes for male or female gonad differentiation that are able to become an MSD gene (for example, *Nr5a1* for GGA 17, *Sox3* for GGA 4p, or *Dmrt1* for GGA Z). It is of note that all members of the gonad differentiation genetic network do not have the same probability to become an MSD gene. For instance, *Sox9*, an important member of the male pathway and a direct target of SRY in mammals, also plays a major role in skeletal development. In humans, its mutation causes a severe skeletal dysplasia called campomelic dysplasia. Its recruitment as an MSD gene was thus hampered by the deleterious side-effects it would have caused. This is the likely explanation for why GGA 18, bearing *Sox9*, appears only once in Table S1. Moreover, we want to emphasize that *Cbx2* also localizes to GGA 18, and in our opinion seems a better MSD candidate than *Sox9*. 

Regardless of why the same genomic regions are repeatedly co-opted to evolve into sex chromosomes, this phenomenon offers the possibility to compare the evolution of a given region in two independent sex-chromosome differentiation events. Rovatsos and Kratochvíl took this opportunity to test the role of the genetic background in the evolution of dosage compensation [104]. In anole lizard (*A. caroliniensis*), X and Y chromosomes evolved from a genomic region homologous to GGA 15. In both males and females, X-linked genes maintain their ancestral expression levels through a male specific two-fold X chromosome expression up-regulation, restoring the ancestral expression level [283]. Thus, complete gene dosage compensation occurs in this species. The same genomic region homologous to GGA 15 was also selected to become Z and W chromosomes in softshell turtles from the family Trionychidae. Rovatsos and Kratochvíl looked for the presence or absence of dosage compensation in *A. ferox* and found that the expression level of Z-linked genes in females is roughly half of that in males. This absence of global gene dosage compensation in the softshell turtle contrasts with that observed in anole lizard, and led the authors to conclude that the apparition of dosage compensation mechanisms is independent of the genomic background. It is clear that genetic background alone does not explain the appearance or absence of dosage compensation, but to conclude that genomic background is of little importance is perhaps a little too radical for several reasons. The appearance of gene dosage compensation is thought to be mostly driven by the presence of haploinsufficient genes which need to be sufficiently expressed in the heterogametic sex. However, gene dosage compensation is only one way among others to achieve this goal: the haploinsufficient gene can also be conserved as a gametolog on the Y or W chromosome, or be translocated to an autosome. Both the loss of a gametolog or the translocation of a haploinsufficient gene are stochastic events which occur by chance and may be selected or not. As turtles and lizards diverged more than 250 Myrs ago, there was enough time for differential loss, conservation or translocation of a few important haploinsufficient genes, which may be sufficient to explain the difference of gene dosage conservation in the two groups today. We thus need the complete list of genes in sex chromosomes of both species, those common to both species, those conserved only in one species, and their status regarding haploinsufficiency before drawing any definitive conclusions. Another point to take into consideration is the variability of the sensitivity to haploinsufficiency between species, which may slightly vary. Such a phenomenon is observed in mammals where, for instance, mutations in *Sox9* or *WT1* produce a phenotype when heterozygous in humans, but only when homozygous in mouse [151,312,313,314,315]. Other comparisons between reptilian species having co-opted the same genomic regions as sex chromosomes are thus necessary. The case of the two turtles, *S. crassicollis* and *G. insculpta*, which both have a XY system homologous to GGA 5 and diverged more recently (80 Myrs), is especially attractive.

### 3.2. What Is Still to Be Discovered and How It Could Be Carried Out

The forty-five clades in Table S1 constitute a minimal estimation of sex-chromosome variation in reptiles, because it is not known if Gymnophthalmidae and Teiidae share the same XY system, and the same uncertainty exists for the genera *Gehyra*, *Dixonius* and *Heteronotia*, and other species marked with a “?”. As described previously, some groups such as Dibamidae, Cordylidae, Teiidae or Amphisbenia have been poorly studied and certainly contain new sex chromosome systems. Other groups such as non-caenophidian snakes, geckos (especially in Gekkonidae family) or chameleons are already known to possess more than one sex determination system, but only a small percentage of their species have been investigated. Future studies by modern cytogenetics or molecular methods will certainly reveal a more diverse variability of sex chromosome systems in these groups. As seen in the first part of this review, modern cytogenetics with CGH or rRNA FISH experiments allow the identification of XY or ZW chromosomes, whereas molecular methods such as genome coverage analysis between male and female and RAD-seq experiments identify the syntenic genomic region which became sex chromosomes. However, genome coverage analysis gives only access to the gene content of X or Z chromosomes, whereas validated RAD-seq markers are only Y- or W-specific. So, how to go further and identify MSD gene in reptiles?

Ideally, a candidate gene must meet four criteria before being declared an MSD gene: (1) it must be localized in the sex-specific part of the sex chromosome, (2) it must be expressed in the gonads at the right period (i.e., at least in the undifferentiated gonad just before the first histological signs of differentiation), (3) its inactivation in one sex must lead to complete sex reversal, and (4) its overexpression in the other sex must cause the opposite sex reversal. Until recently, these last two functional criteria could not be satisfied in reptiles. However, the establishment of a lentivirus-mediated RNAi gene-modulating method through injection of turtle eggs [316] and the establishment of transgenic lizards by CRISPR-Cas9 gene editing through microinjection of unfertilized oocytes [317] open the way for functional genetic studies in reptiles. We want to stress that all four criteria are important and that the two functional criteria alone are not sufficient. In the softshell turtle *P. sinensis* for instance, there is masculinization of the ZZ embryos overexpressing *Dmrt1* and feminization of the ZZ gonads following *Dmrt1* knockdown [216]; however, the *Dmrt1* gene localizes to chromosome 4 [91] and not to the sex minichromosome. Moreover, *Amh* overexpression or loss of function gives the same phenotype as for *Dmrt1* [318]. Therefore, at least two genes meet the two functional criteria, but none of them is the MSD gene. In order to meet the four criteria, many animals are needed, and this is a major drawback when the species is rare or does not reproduce in captivity. Several methods using next-generation sequencing data, can be used to circumvent this problem (see [310] for review). One of them is a male-female transcriptomic [216] or transcriptome/genome [283] subtraction approach allowing the identification of Y- or W-linked transcripts. This direct identification of gametologs, coupled with the measure of their Ks, leads to a very short list of candidate MSD genes. A major caveat of this approach is its inability to identify the MSD gene when the MSD gene is not a gametolog but acts instead in a dose-dependent manner as a male gene on the Z chromosome or a female gene on the X chromosome. This seems to be the case in caenophidian snakes, trionychid turtles or lacertid lizards, for example. In the case of trionychid turtles, two groups identified 100–120 X-linked genes devoid of SNPs as potential candidate MSD genes in the genus *Apalone* [104,105]. There are two ways to condense this list. The first transcriptional approach consists of mining the RNA-seq data to discard every gene not expressed in gonads or expressed at similar levels in gonads of both sexes at the time of gonad differentiation. The second genomic approach is to sequence the genome of the most divergent species sharing the same sex chromosome system, in the hope that the speed and trajectory of W degeneration was different enough, which should produce a slightly different list of candidate genes for comparison. In the case of trionychids, a *Cycloderma* species which belongs to the other subfamily could be a good choice. It is likely that MSD genes acting in a dose-dependent manner will be much more difficult to identify than MSD genes which are gametologs. For this latter category, the recent advances in high-fidelity long-read sequencing have made these techniques, combined with Hi-C, powerful enough to produce the sequence of both sex chromosomes by sequencing only the heterogametic sex. It is thus the method of choice for rare or endangered species.

## 4. Conclusions

There is no doubt that the identification of the first MSD gene in reptiles will be made in the near future. Today the best candidate is *PPP1CCY* (Protein phosphatase 1, catalytic subunit gamma isozyme) in Pleurodonts [283]. Its inactivation or overexpression in the anole lizard is now technically feasible [317] and could be tested soon. Another good candidate is *PPP1R12A* (Protein Phosphatase 1 Regulatory subunit12A) in skinks [217]. Up to now there are no transgenic skinks, but some skinks are relatively easy to breed in captivity and there is no reason the technique of transgenesis by CRISPR-Cas9 gene editing through microinjection of unfertilized oocytes could not be implemented in skinks. Knowledge of sex-linked genes of trionychids is currently the most advanced in turtles. However, the likely dose-dependent nature of its MSD gene impairs its chance to be identified and tested by knock-down experiments. The study of turtle sex chromosomes may unveil some candidates to be tested in chelids or *S. crassicollis*. The decreasing cost of high-fidelity long-read sequencing will make it easier to obtain more numerous and more complete sequences of reptilian sex chromosomes. Their study and comparison will shed light on the evolutionary dynamics of sex chromosomes, give clues as to why so many ancient reptilian sex chromosomes still are homomorphic, and help to explain why global gene dosage compensation mechanisms are only found in certain lineages. However, as recently emphasized [319], high-quality genomes are only a starting point to understanding sex evolution and must be completed by an integrative approach to achieve a complete understanding of the sexome in all its complexity.

## Data Availability

Not applicable.

## Supplementary Materials

The following are available online at https://www.mdpi.com/article/10.3390/genes12111822/s1, Table S1: Sex-chromosome variation in reptiles.
